# Transfer learning enables the molecular transformer to predict regio- and stereoselective reactions on carbohydrates

**DOI:** 10.1038/s41467-020-18671-7

**Published:** 2020-09-25

**Authors:** Giorgio Pesciullesi, Philippe Schwaller, Teodoro Laino, Jean-Louis Reymond

**Affiliations:** 1 Department of Chemistry and Biochemistry, University of Bern, Freiestrasse 3, 3012 Bern, Switzerland; 2 IBM Research—Europe, Säumerstrasse 4, 8803 Rüschlikon, Switzerland

**Keywords:** Cheminformatics, Carbohydrate chemistry, Natural product synthesis

## Abstract

Organic synthesis methodology enables the synthesis of complex molecules and materials used in all fields of science and technology and represents a vast body of accumulated knowledge optimally suited for deep learning. While most organic reactions involve distinct functional groups and can readily be learned by deep learning models and chemists alike, regio- and stereoselective transformations are more challenging because their outcome also depends on functional group surroundings. Here, we challenge the Molecular Transformer model to predict reactions on carbohydrates where regio- and stereoselectivity are notoriously difficult to predict. We show that transfer learning of the general patent reaction model with a small set of carbohydrate reactions produces a specialized model returning predictions for carbohydrate reactions with remarkable accuracy. We validate these predictions experimentally with the synthesis of a lipid-linked oligosaccharide involving regioselective protections and stereoselective glycosylations. The transfer learning approach should be applicable to any reaction class of interest.

## Abstract (web summary)

Organic reactions can readily be learned by deep learning models, however, stereochemistry is still a challenge. Here, the authors fine tune a general model using a small dataset, then predict and validate experimentally regio- and stereo-selectivity for various carbohydrates transformations.

## Introduction

Organic synthesis is a complex problem-solving task in which the vast knowledge accumulated in the field of organic chemistry is used to create new molecules, starting from simple commercially available building blocks^1^. Because of its complexity, organic synthesis is believed to be one of the main bottlenecks in pharmaceutical research and development^2^, and having accurate models to predict reaction outcome could boost chemists’ productivity by reducing the number of experiments to perform.

Machine learning has long been present in the chemical domain, tackling challenges than range, for example for quantitative structure–activity relationship predictions^3^, virtual screening^4^ and quantum chemistry^5,6^. Enabled by algorithmic advances in deep learning^7–10^ and the availability of large reaction data sets^11,12^, reaction prediction methods have emerged in recent years^13–22^. Those reaction prediction methods can be divided into two categories^23^, bond change prediction methods using graph neural networks^14,16–18,22^ and product SMILES generation using sequence-2-sequence models^15,19^.

Reaction prediction tasks are typically evaluated on the USPTO_MIT benchmark^14^, which does not contain molecules with defined stereocenters. Currently, the best prediction algorithm in terms of performance is the Molecular Transformer^10,19^. The architecture is based on the ground-breaking work by Vaswani et al.^10^, which revolutionised the field of neural machine translation, where sentences in one language are translated into another language. In contrast, for reaction prediction, the model learns to translate the precursors’ Simplified molecular-input line-entry system (SMILES)^24^ representation into the product SMILES.

The Molecular Transformer can be accessed for free through the IBM RXN for Chemistry platform^25^. Compared to other methods, such as graph neural networks-based ones, the advantages of the Molecular Transformer approaches are that they do not require mapping between the product and reactant atoms in the training^26^ and inputs can contain stereochemistry. In fact, sequence-2-sequence approaches, like the Molecular Transformer^10,19^, are currently the only large-scale reaction prediction approaches capable of handling stereochemistry. Stereochemistry is systematically avoided in graph-based methods, as the connection table and adjacency matrix of two stereoisomers is identical. Although stereoselectivity can theoretically be predicted by the Molecular Transformers^19^, it is one of their most significant weaknesses because of the lack of clean training data. To date, their performance on predicting specific stereochemical reactions has not been investigated.

In this work, we investigate the adaptation of the Molecular Transformer to correctly predict regio- and stereoselective reactions. As study case we focus on carbohydrates, a class of molecules for which the stereochemistry and the high degree of functionalization are key reactivity factors. Carbohydrate chemistry is essential for accessing complex glycans that are used as tool compounds to investigate fundamental biological processes such as protein glycosylation^27–29^, as well as for the preparation of synthetic vaccines^30–32^. Predicting the outcome of carbohydrate transformations, such as regioselective protection/deprotection of multiple hydroxyl groups or the stereospecificity of glycosylation reactions, is a very difficult task even for experienced carbohydrate chemists^33,34^, implying that this field of research might particularly benefit from computer-assisted reaction prediction tools.

First, we investigate transfer learning with a specialized subset of reactions as a means to adapt the Molecular Transformer to achieve high performance on carbohydrate reactions. Transfer learning, where a model is trained on a task with abundant data and either simultaneously trained or subsequently fine-tuned on another task with less data available^35^, has recently led to significant advancements in Natural Language Processing^36–39^. For instance, it has been used to improve translation performance in low-resource languages^36^. More recently, unsupervised pretraining transfer learning strategies have successfully been applied to sequence-2-sequence models^37,40^. In the chemical domain, transfer learning has enabled the development of accurate neural network potential for quantum mechanical calculations^41^ and shows great potential to solve other challenges^42^. For transfer learning we use a set of 20k carbohydrate reactions from the literature, comprising protection/deprotection and glycosylation sequences. We explore multitask learning, as well as sequential transfer learning, and show that the adapted model, called the Carbohydrate Transformer, performs significantly better than the general model on carbohydrate transformations and a model trained on carbohydrate reactions only.

Second, we perform a detailed experimental assessment of the deep learning reaction prediction model and test the Carbohydrate Transformer on unpublished reactions. Our assessment consists of a 14-step total synthesis of a modified substrate of a eukaryotic oligosaccharil transferase (OST). We also challenge our Carbohydrate Transformer to predict the reactions from the recently published total syntheses of the trisaccharide of *Pseudomonas aeruginosa* and *Staphylococcus aureus*^43^ as a further assessment on more complex carbohydrate reactions. Those reactions would be considered challenging to predict, even for carbohydrate experts.

Overall, we observe a consistent top-1 prediction accuracy above 70%, which roughly means a 30% increase compared to the original Molecular Transformer baseline. We find that the confidence score is a good predictor of prediction reliability and that many wrong predictions have chemical reasons such as the lack of reagent stoichiometry in the training data. The approach we used to learn carbohydrate reactions could be applied to any reaction class. Hence, it is expected to have a significant impact on the field of organic synthesis, as models like the Molecular Transformer^19^ can easily be specialized for the reaction subspaces that individual chemists are most interest in.

## Results

### Data availability scenarios

Besides the additional complexity, the main challenges for learning to predict stereochemical reactions is the data. In the largest open-source reaction data set by Lowe^11,12^, which fueled the recent advancements in machine learning for chemical reaction prediction, stereochemistry, and specifically reactions involving carbohydrates are underrepresented and of poor quality. Hence, those reactions are problematic to learn.

In this work, we explore two real world scenarios, where there exist a large data set of generic chemical reactions and a small data set of complex and specific reactions. In our case, we use a data set derived from the US patent reactions by Lowe^12^ as the large data set containing 1.1M reactions. We call this data set USPTO. For the specific reaction, we chose carbohydrates reactions, but the methods described could be applied to any reaction class of interest. We manually extracted reactions from the Reaxys^44^ database, selected from papers of 26 authors in the field of carbohydrate chemistry. The small data set of 25k reactions will be referred to as CARBO for the remainder of the publication. We split the USPTO and the CARBO data set into train, validation and test sets. The reaction data was canonicalised using RDKit^45^. A more detailed description of the data is found in Supplementary Note 1.

If the access to the large and small sets is given, the two data sets can be used simultaneously for training. We call this first scenario multitask. However, depending on the situation, direct access to the data of the generic data set may not be possible. For example, a company A may have proprietary reaction data precluded from external sharings. Company A could still train a model using their own data and share their model without revealing the exact data points. The trained model extracts some general chemical reactivity knowledge and could be shared without exposing company proprietary information. This pretrained model could then serve as a starting point to further train the model on another source of reactions. We call this scenario fine-tuning.

**Fig. 1:**
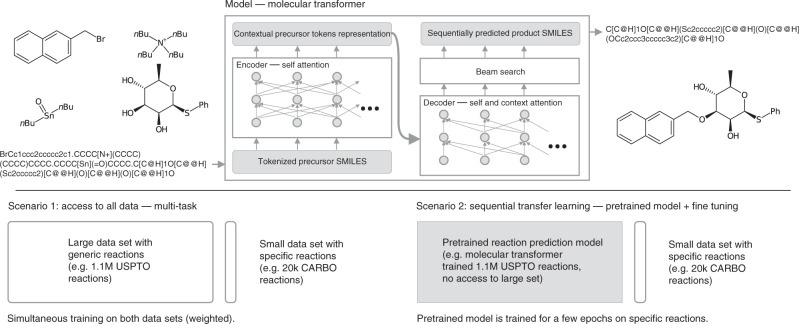
Molecular Transformer model and data scenarios. Sequence-2-sequence prediction of carbohydrate reactions and the two transfer learning scenarios, namely, multitask and sequential training.

A visualisation of the model and the two scenarios can be found in Fig. 1.

**Fig. 2:**
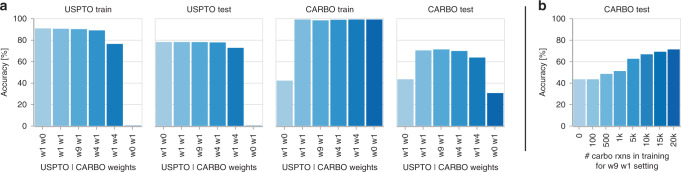
Multitask scenario results. **a** Top-1 accuracy of models trained with different weights on the USPTO and CARBO data set (the first number corresponds to the weight on the USPTO data set and the second to the weight on the CARBO data set). **b** Top-1 accuracy for a model trained in the weight 9 weight 1 setting, where the number of reactions in the CARBO data set was reduced. Source data are provided as a Source Data file.

In the multitask scenario, we investigated different reaction weighting schemes between the two sets. A comparison of the top-1 accuracies on the USPTO train, USPTO test, CARBO train and CARBO test sets for models trained with different weights for the USPTO train and CARBO train sets are shown in Fig. 2a. The weights describe in what proportion reactions from the two sets are shown per training batch. For example, weight 1 on USPTO and weight 1 on CARBO means that for one USPTO reaction one CARBO reaction is shown. As can be seen in the Figure, the highest accuracy on the CARBO test set (71.2 %) is obtained with weight 9 on the USPTO set and weight 1 on the CARBO set (w9w1). As expected, training only with the CARBO train set leads to a poor CARBO test set accuracy (30.4%). As 20k reactions are not enough for the model to learn predict organic chemistry. The accuracy reached by the model trained purely on the USPTO data reaches 43.3%. It therefore performs better than the model trained purely on the CARBO reactions. In Fig. 2b, we assess the effect of the size of the CARBO train set. The accuracy continuously increases from 43.3 to 71.2% with an increasing number of reactions in the train set.

**Fig. 3:**
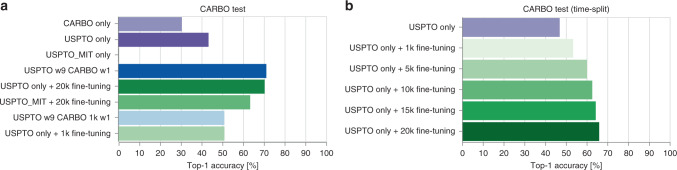
Fine-tuning scenario results. **a** CARBO random split test set performance for different training strategies. In green are the top-1 accuracies of the models that were fine-tuned on either 1k or 20k CARBO reactions shown. For comparison, we included in purple the top-1 accuracies of the models trained on the single data sets (CARBO, USPTO, and USPTO_MIT). Blue are the performances of models trained in the multitask scenario. **b** CARBO time split test set performance for different fine-tuning set sizes. Source data are provided as a Source Data file.

For the fine-tuning scenario, where access to the large generic data set is not given but a model, pretrained on the large data set, is available instead, the results on the CARBO and USPTO test sets are shown in Fig. 3a. After training the model on the CARBO train set, the top-1 accuracy reaches a 70.3%, similar to the model that was trained on the two data sets simultaneously. The observed behavior is the same when less CARBO reactions are available. Also for 1k CARBO reactions, the fine-tuning model matched the accuracy of the corresponding multitask model.

For this scenario, we analysed the effect of the train, validation, and test split in more detail. We compared the random split described above to a time split, where we included CARBO reactions first published before 2016 into the train and validation sets and the reactions published from 2016 into the test set (2831 reactions). We investigated different fine-tune set sizes (1k, 5k, 10k, 15k, and 20k). As seen in Fig. 3b, compared to the random split the top-1 accuracy with the 20k fine-tuning dropped slightly to 66% but it is still substantially larger than the accuracy that could be obtained with the generic USPTO training set only. Already with 5k CARBO reactions, an accuracy above 60% was reached. The larger the CARBO fine-tuning set, the better the performance of the fine-tuned model.

Besides the fact that the reactions in the large data set do not need to be revealed, another advantage is the short fine-tuning training time. The fine-tuning requires only 5k steps compared to 250k steps in the multitask scenario. However, if time and access to both data sets are given, it is better to train simultaneously on all data for a longer time as the performance on the large data set does not decrease, as it does in the fine-tuning scenario. If the interest is only in a specific reaction class, short adaptation times or if generic data is not available, then fine-tuning a pretrained model is better.

To further demonstrate the effectiveness of the fine-tuning approach, we performed an experiment where we pretrained a model on a data set without stereochemical information. To do so, we used the USPTO_MIT data set by Jin et al.^14^. As seen in Fig. 3a, although the pretrained model does not manage to predict any CARBO test set reactions, after fine-tuning for 6k steps the model reaches an accuracy of 63.3%. The accuracy was not as high as with USPTO pretraining but a significant improvement over the 0.0% correctly predicted reactions by the pretrained model. The low accuracy after pretraining was expected as none of the chiral center tokens (e.g. “[C@H]”, “[C@@H]”) were present in the training set. The fine-tuning result shows that the Molecular Transformer model is able to learn new concepts within a few thousands training steps on 20k data points.

In the next sections, we will compare the model trained only on the USPTO data, which was also used as pretrained model (USPTO model) with the model that was then fine-tuned on the 20k CARBO reactions (CARBO model).

### Experimental assessment

**Fig. 4:**
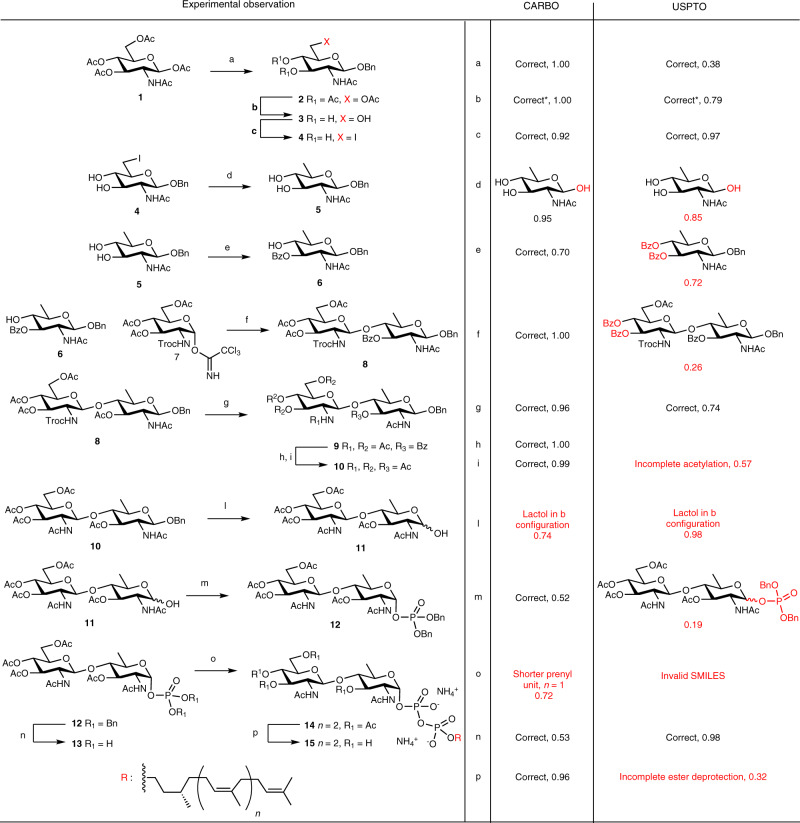
Synthesis of lipid-linked oligosaccharide (LLO). Reaction conditions: **a** BnOH, Yb(OTf)_3_, DCE, 90 °C, 2h, 78%. **b** MeONa, MeOH, sonication, 30 min. **c** PPh_3_, I_2_, imidazole, THF, 1h, reflux, 88% over two steps. **d** Pd/C, NH_4_OH, H_2_, THF/H_2_O, 30 min, 77%. **e** BzCl, pyr,  −35°, 70%. **f** BF_3_Et_2_O, 4 MS, DCM, 26 h, 73%. **g** Zn, Ac_2_O, AcOH, DCE 50°, 3 h, 96%. **h** MeONa, MeOH/DMF, 4 days. **i** Ac_2_O, 4-(Dimethylamino)pyridine, pyr, 76% over three steps. **l** H_2_, THF/H_2_O, 10 bar, 16 h. **m** LiHMDS, tetrabenzylpyrophosphate, 53%. **n** H_2_, THF/MeOH, 1 h. **o** farnesylnerol, CDI, DMF, then **11**, 5 days, 18%. **p** MeOH, NH_4_OH, 16 h, qte. An asterisk represents “*” reaction present in the training set.

Although the accuracy of the transformer has been widely assessed^19^, an experimental validation is still missing. Here, we decided to validate both the transformer and the augmented precision of the CARBO model on a recently realized synthetic sequence from our own laboratory, absent from the training data. This sequence is a 14-step synthesis of lipid-linked oligosaccharide (LLO) **15** to be used as a substrate to study OST^46,47^ (Fig. 4). The sequence contains typical carbohydrate chemistry: protecting group manipulations (steps: b, h, i, l n, p), functional group manipulations (step c, d), regioselective protections (step e), a *β*-selective glycosylation (step g) and an *α*-selective phosphorylation (step m). The latter regio- and stereoselective transformations are of particular interest because their selectivity is generally difficult to control and to predict, even for experienced synthetic chemists.

We used both the general USPTO model and the fine-tuned CARBO model to predict 13 of the 14 steps in the sequence (step b was removed since it appeared in the training set). The USPTO only made four correct predictions (31%), which were either standard protecting group manipulations (step a, g, n) or functional group exchanges (step c). The CARBO model also correctly predicted these four simple reactions, but additionally, made another six correct predictions, including the regioselective benzoylation (**5**–**6**, step e) and the *β*-selective phosphorylation (**11**–**12**, step m), corresponding to a 77% success rate and a 46% improvement over the USPTO model, in line with the overall statistics presented above.

In detail, the CARBO model only made three mistakes. The first one concerns the reduction of the primary iodide **4** to a methyl group in **5** by hydrogenation, which is mistakenly predicted to also reduce the benzyl glycoside. The USPTO model makes the same mistake. Both models have not learned that carrying out the reaction in the presence of ammonia reduces the catalyst activity and avoids debenzylation, as no such reaction was present in the training sets. The second mistake concerns a similar reduction of the benzyl glycoside in **10** (step l), which is predicted to yield the *β*-lactol while the product **11** is in fact formed as an anomeric mixture. Again, the USPTO model makes the same mistake. Both models ignore that the initially formed *β*-lactol equilibrates spontaneously to the anomeric mixture via ring opening. Finally, the CARBO model predicts a shortened prenyl chain in the phosphate coupling reaction forming the protected LLO **14** (step o), which does not make chemical sense. In this case it should be noted that the CARBO training set does not contain a single LLO molecule, and that the USPTO model performs worse since it returns an invalid SMILES for this reaction.

**Fig. 5:**
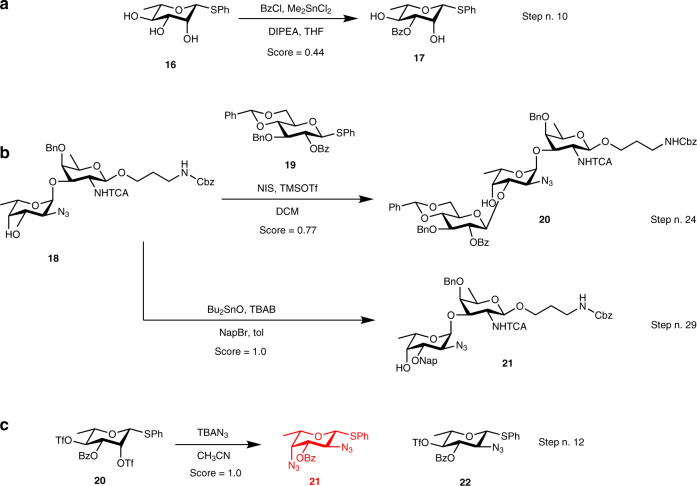
Reactions predicted from recent literature. **a**, **b** Reactions correctly predicted. **c** wrongly predicted reaction (red structure) due to missing reagent stoichiometry in the model: only one equivalent of NaN_3_ was used resulting in single substitution, while the model predicts double substitution.

We obtained similar prediction performances from both models when analyzing a recently published total syntheses of the trisaccharide repeating unit of *Pseudomonas aeruginosa* and *Staphylococcus aureus*^43^. Those synthetic sequences comprises four difficult regio- and stereoselective glycosylation steps and five regioselective protection steps that are of particular interest. Out of the 38 reactions that are absent from the training set in this sequence (Supplementary Figs. 2–7), the USPTO model predicts only 15 reactions (39%) correctly, and none of the difficult steps mentioned above. The CARBO model performs much better and correctly predicts 26 of the 38 reactions, corresponding to a 68% overall accuracy and a 29% gain over the USPTO model. In particular, the CARBO model correctly predicts the regioselectivity of the dimethyltin chloride mediated benzoylation of L-Rhamnopyranoside **16** (step no. 10), the difficult regio- and stereoselective glycosylation at position 3 of the terminal fucosyl in disaccharide **18** (step no. 24) as well as the regioselective protection of the same disaccharide at position 3 (step no. 29), all of which are nonobvious even for synthetic chemists (Fig. 5). Interestingly, the CARBO model predicts a double substitution of bis-triflate **19** instead of the correct single substitution at position 2, which the USPTO model correctly predicts. In this case it should be noted that the outcome of the reaction is dictated by stoichiometry (only one equivalent of the azide nucleophile), an information which is absent from the training data. In contrast to the USPTO training set, that contains only single azide substitutions, the CARBO training set contains single, as well as double substitutions. An analysis of the stereo centres in both data sets can be sound in Supplementary Table 1 and Supplementary Fig. 1.

**Fig. 6:**
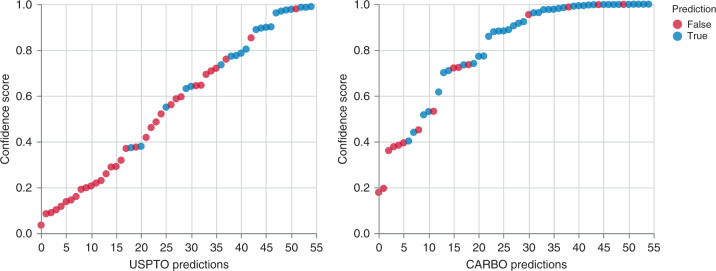
Analysis of prediction confidence scores. Predictions (ordered by confidence score) for the experimental assessment. Source data are provided as a Source Data file.

Every predicted reaction is associated with a confidence score^19^, which is calculated from the product of the probabilities of the predicted product tokens. Interestingly, the confidence score correlates with the correctness of the prediction (Fig. 6). For both models most of the correct predictions have a score higher than 0.8.

To have a closer look at the capabilities of the model to self-estimate its own uncertainty, we analyzed every reaction in detail. In some cases, we observe epimerization or rearrangements that have little chemical significance and are associated with low score values. This even occurs in more trivial transformations, such as amine acetylation of the trisaccharide in reaction 27 (scheme S3). Although the model is not able to predict the correct product, its low score seems to indicate that the model senses its own mistake. The second class are arguably wrong predictions that have high confidence for chemical reasons. Such an example is the previously discussed reaction 12 (Scheme 2, entry c) whose outcome is influenced by stoichiometry that together with other reaction conditions, is excluded from the training data, making these reactions extremely difficult to predict.

Similar to previous work^19^, one of the limitations of current SMILES-2-SMILES models is that environmental reaction conditions like temperature and pressure are not taken into account. Those conditions are often missing in the data sets, and even if present, it would not be straightforward to codify temperature profiles applied during chemical reactions. Another limitation is the data coverage and quality. As pointed out above, most of the wrong predictions can be explained with the data that the models have seen during training.

The availability of large high-quality open-source reaction data set containing information detailed on amounts, stoichiometry, and reaction conditions could substantially improve reaction prediction models.

## Discussion

In this work, we demonstrated that transfer learning can be successfully applied to a generally trained transformer model using as few as 20k data points to derive a specific model that predicts reactions from a specific class with significantly improved performance. Transfer learning of the general molecular transformer model, trained on the USPTO data set to a specific set of reactions, to obtain a high-performance specialized model as demonstrated here should be generally applicable towards any subclass of specific reactions of interest.

Here we used transfer learning to improve predictions of regio- and stereoselectivity, a central aspect of synthetic chemistry that has not been systematically evaluated previously by reaction prediction models, in part due to the fact that the Molecular Transformer is currently the only model able to handle stereochemistry. As a test case we examined carbohydrates, a well-defined class of molecules for which reactions are difficult to predict even for experienced chemists, and subjected our model to experimental validation. We anticipate that the Carbohydrate Transformer will serve the practical purpose of improving the efficiency of complex carbohydrate syntheses. The model can guide chemists by predicting and scoring potential carbohydrates reactions before performing them experimentally. The fact that the confidence score correlates with prediction accuracy offers a simple metric to judge the quality of predictions. The shortcomings noted should be addressable by extending the training set with reactions that are not predicted well.

## Methods

### Reaction prediction model

All the experiments in this work were run with the Molecular Transformer model^19^, which is illustrated in Fig. 1. For details on the architecture we refer the reader to^10,19^. We used Pytorch^48^ and the OpenNMT^49^ framework to build, train and test our models. Hyperparameters and a detailed description of the data sets can be found in the supplementary information. The investigated task is reaction prediction, where the aim is to predict the exact structural formula, including stereochemistry, of the products that are formed from a given a set of precursors as input. In the inputs, no difference is made between reactant and reagent molecules^19^. Following previous work^13,15,19^, we use accuracy as the evaluation metric. The reported accuracies describe the percentage of correct reactions. A reaction is counted as correct only if the predicted products exactly matches the products reported in the literature after canonicalisation using RDKit^45^. The canonicalisation is required as multiple SMILES can represent the same molecule.

### Chemical synthesis

All reagents were purchased from commercial sources and used without further purifications unless otherwise stated. All reactions were carried out in flame-dried round-bottomed-flask under an argon atmosphere, except if specified. Room temperature (rt) refers to ambient temperature. Temperatures of 0 °C were maintained using an ice-water,  −78 °C with acetone/dry ice bath and the other temperatures using a cryostat. Dry solvents were obtained by passing commercially available pre-dried, oxygen-free formulations through activated alumina columns. Hydrogenation was performed at room pressure using H_2_ filled balloon. Chromatographic purifications were performed with silica gel pore size 60, 230–400 mesh particle size (Sigma-Aldrich). Thin layer chromatography was performed using ALUGRAM Xtra Sil G/UV on pre-coated aluminium sheets, using UV light as a visualizing, and a basic aqueous potassium permanganate solution and ceric ammonium molybdate as developing agents. NMR spectra for ^1^H, ^13^C, DEPT, ^31^P, COSY, HSQC, HMBC, and NOE were recorded at rt with a Bruker AV (400 MHz ^1^H). Spectra were and processed using TopSpin 3.6.1 software. Chemical shifts are reported in *δ* (ppm) relative units to residual solvent peaks CDCl_3_ (7.26 ppm for ^1^H and 77.2 ppm for ^13^C) and MeOD (3.31 ppm for ^1^H and 49.00 ppm for ^13^C). Splitting patterns are assigned as s (singlet), d (doublet), t (triplet), q (quartet), quint (quintet), multiplet (m), dd (doublet of doublets), and td (triplet of doublets). High-resolution mass spectra was provided by the “Service of Mass Spectrometry” at the Department of Chemistry and Biochemistry in Bern and were obtained by electron spray ionization in positive or negative mode recorded on a Thermo Scientific LTQ Orbitrap XL. For the experimental procedures, NMR spectra and physical data of compounds 2–15, see Supplementary Note 3.

## Supplementary information

Supplementary Information

## Data Availability

The USPTO data set derived from Lowe^12^ that we used for training and evaluation, our carbohydrate reactions, as well as the ones from the work of Behera et al.^43^ are available from (https://github.com/rxn4chemistry/OpenNMT-py/tree/carbohydrate_transformer). Source data are provided with this paper.

## Source data

Source Data

## Code availability

The code and trained models are available from (https://github.com/rxn4chemistry/OpenNMT-py/tree/carbohydrate_transformer). The models are compatible with OpenNMT-py^49,50^, which was used for training and evaluation. The SMILES tokenization function for preprocessing the inputs is found on the Molecular Transformer repository^19,51^. The setup and hyperparameters can also be found in Supplementary Note 2.
