# Supplementary material for: Transfer learning enables the molecular transformer to predict regio- and stereoselective reactions on carbohydrates
Source: Nat Commun. 2020 Sep 25;11:4874. doi: 10.1038/s41467-020-18671-7 (PMC7519051; doi:10.1038/s41467-020-18671-7)
Supplement: Supplementary file 1 — Supplementary Information [file 41467_2020_18671_MOESM1_ESM.pdf]

Supplementary Information - Transfer Learning Enables the Molecular Transformer to Predict Regio- and Stereoselective Reactions on Carbohydrates

Pesciullesi, Schwaller et al.*

E-mail: jean-louis.reymond@dcb.unibe.ch

Supplementary Note 1: Data

Recent advancement in machine learning for reaction prediction were made possible thanks to the vast availability of chemical reaction data. The largest open-source reaction data set was constructed by Lowe[1] and subsequently filtered and cleaned by different groups.[2–4] A general overview of the different reaction data sets can be found in.[5] To have a large set covering a broad range of chemical reaction classes, we started from the raw data of Lowe and constructed the reaction smiles from the extracted components. We filtered out all reactions for which we cannot match all the components to a SMILES structure. For instance, if the metal catalyst in a Suzuki coupling reaction could not be mapped to a structure because of a wrong IUPAC name in the patent, the reaction was tagged as incomplete and removed. After canonicalising the reactions using RDKit[6] and removing duplicates the generic data set (USPTO) yielded 1.2M reaction smiles. We split the data into training, validation and test sets (1.09M / 0.6M /0.6M), making sure that same products remained in the same set. Trained models and our reaction data can be found on `https://github.com/rxn4chemistry/OpenNMT-py/tree/carbohydrate_transformer`.

The second data set we use in this work is specific to carbohydrates chemistry. We manually extracted reactions from papers of 26 authors in the field of carbohydrate chemistry using Reaxys.[7] We considered full reactions with preparation and filtered out multi-step and enzymatic reactions. Reagents, solvents and catalysts, for which in the Reaxys database only the chemical names are available, were converted to SMILES structures and added to the precursors in the reaction SMILES. We only kept reactions, for which we could convert all relevant names to SMILES. We removed reactions with multiple products, the reactions without stereocenter in the product and those with just a single precursor. After the removal of duplicate reactions, the carbohydrate reactions data set (CARBO) yielded 25k reaction smiles. Similar as for the USPTO data set, we split the data into training, validation and test sets (19.7k / 2.4k / 2.5k). We also make sure that all reactions resulting in the same product molecule are in the same set. On average the products contained 6.4 stereo centers.

The following is the list of the 50 most commonly appearing authors in our Carbo data set (ordered alphabetically, the ones used for the query are highlighted with a star): Ando, Hiromune*; Bandiera, Tiziano; Beau, Jean-Marie*; Bernet, Bruno; Bertozzi, Carolyn R.*; Bertozzi, Fabio; Bols, Mikael*; Boons, Geert-Jan*; Cheng, Ting-Jen R.; Crich, David*; Davis, Benjamin G.*; Demchenko, Alexei V.*; Ernst, Beat*; Fang, Jim-Min; Fujimoto, Yukari; Fukase, Koichi*; Hasegawa, Akira; Hotha, Srinivas*; Hui, Yongzheng; Hung, Shang-Cheng; Imamura, Akihiro; Ishida, Hideharu*; Jung, Karl-Heinz; Kajihara, Yasuhiro*; Kajimoto, Tetsuya; Kiso, Makoto; Kulkarni*, Suvarn S.*; Kusumoto, Shoichi; Li, Qin; Lin, Chun-Cheng; Oscarson, Stefan*; Pedersen, Christian Marcus; Pornsuriyasak, Papapida; Schmidt, Richard R*.; Schwardt, Oliver; Seeberger, Peter H.*; Shie, Jiun-Jie; Stuetz, Arnold E.; Suda, Yasuo; Sun, Jiansong; Urban, Dominique; Vasella, Andrea; Vincent, Stephane P.*; Withers, Stephen G.*; Wong, Chi-Huey*; Xiong, De-Cai; Yang, Jin-Song*; Ye, Xin-Shan*; Yu, Biao*; Zhang, Li-He.

Supplementary Note 2: Hyperparameters and training details

The training and evaluation was performed using OpenNMT-py.[8,9]

Anaconda Environment

To reproduce our results and run our models create the following conda environment:

```
conda create -n carbo python=3.6 -y
conda activate carbo
conda install -c rdkit rdkit=2019.03.2 -y
conda install -c pytorch pytorch=1.2.0 -y
pip install OpenNMT-py==1.0.0.rc2
```

Preprocessing of reactions

Prepare the OpenNMT input files running:

```
onmt_preprocess -train_src $DATADIR/src-train.txt \
    -train_tgt $DATADIR/tgt-train.txt \
    -valid_src $DATADIR/src-valid.txt \
    -valid_tgt $DATADIR/tgt-valid.txt \
    -save_data $DATADIR/preprocessed_onmt36 -share_vocab \
    -src_seq_length 3000 -tgt_seq_length 3000 \
    -src_vocab_size 3000 -tgt_vocab_size 3000
```

The tokenization function, which is used to split the reaction Smiles into tokenized reactions, is available from.[10,11]

Training

We used OpenNMT-py and trained the *multi-task* and single data set models with the following hyperparameters.

```
onmt_train -data $DATADIR/preprocessed_onmt36  \
    -save_model  uspto_MT384 \
    -seed $SEED -gpu_ranks 0  \
    -train_steps 250000 -param_init 0 \
    -param_init_glorot -max_generator_batches 32 \
    -batch_size 6144 -batch_type tokens \
     -normalization tokens -max_grad_norm 0  -accum_count 4 \
    -optim adam -adam_beta1 0.9 -adam_beta2 0.998 -decay_method noam  \
    -warmup_steps 8000 -learning_rate 2 -label_smoothing 0.0 \
    -layers 4 -rnn_size  384 -word_vec_size 384 \
    -encoder_type transformer -decoder_type transformer \
    -dropout 0.1 -position_encoding -share_embeddings  \
    -global_attention general -global_attention_function softmax \
    -self_attn_type scaled-dot -heads 8 -transformer_ff 2048
```

The weights for the data sets can be set using the arguments,

```
-data_ids uspto carbo --data_weights $w1 $w2
```

the weights in what proportion examples from the two data sets are shown within a batch.

For the fine-tuning phase we started from the last checkpoint of the training on the USPTO data set and trained for 6k steps on the CARBO dataset:

```
-train_from /path/to/checkpoint
```

Predicting reaction outcomes

We test our models and predict reactions with a beam size of 5 and a max_length of 300 tokens using the *onmt_translate* script from OpenNMT-py.[8]

```
onmt_translate -model uspto_model_pretrained.pt \
    -src $DATADIR/src-test.txt -output predictions.txt \
    -n_best 1 -beam_size 5 -max_length 300 \
    -batch_size 64
```

Supplementary Tables

Supplementary Table 1: Product stereo centres per reaction statistics in USPTO and CARBO data sets. Statistics on the number of stereo centres in the products of the different reaction data sets. While the USPTO data set has 0.36 stereo centres in the product on average, there are over 6 in the CARBO data set.

	USPTO	CARBO (before 2016)	CARBO (2016 and after)
mean	0.36	6.43	6.24
std	1.01	5.06	5.00
min	0	1	1
25%	0	4	4
50%	0	5	5
75%	0	8	6
max	4	50	39

Supplementary Figures

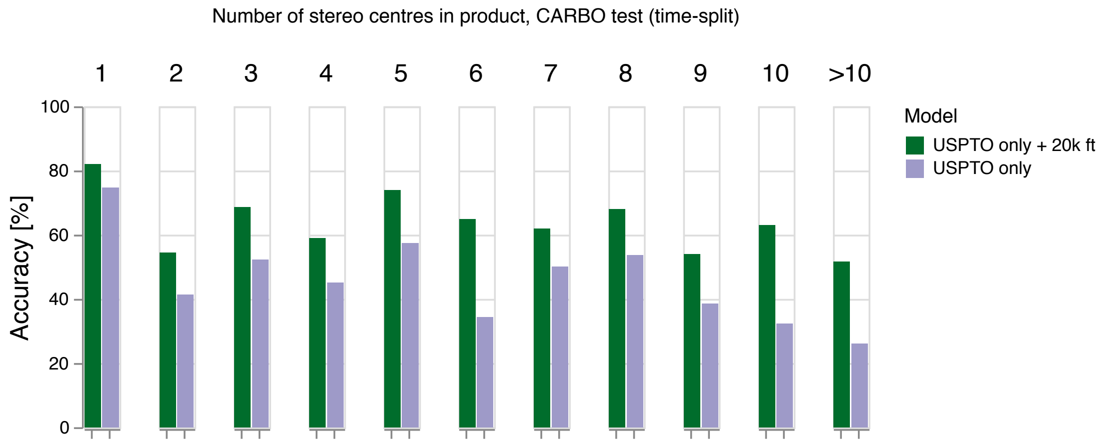

Supplementary Figure 1: Number of stereocenters in product, CARBO test (time-split) Comparison of the performance of the baseline (USPTO only) to the fine-tuned model (USPTO only + 20k ft) on the time-split CARBO test set. The transfer learning increased the accuracy of the models for all the bins. Both models perform the best on reactions that have only one stereo center in the product but there is no clear correlation between the number of stereo centres and the accuracy. The accuracy is divided by the number of stereo centres in the product of the time-split CARBO test set. Source data are provided as a Source Data file.

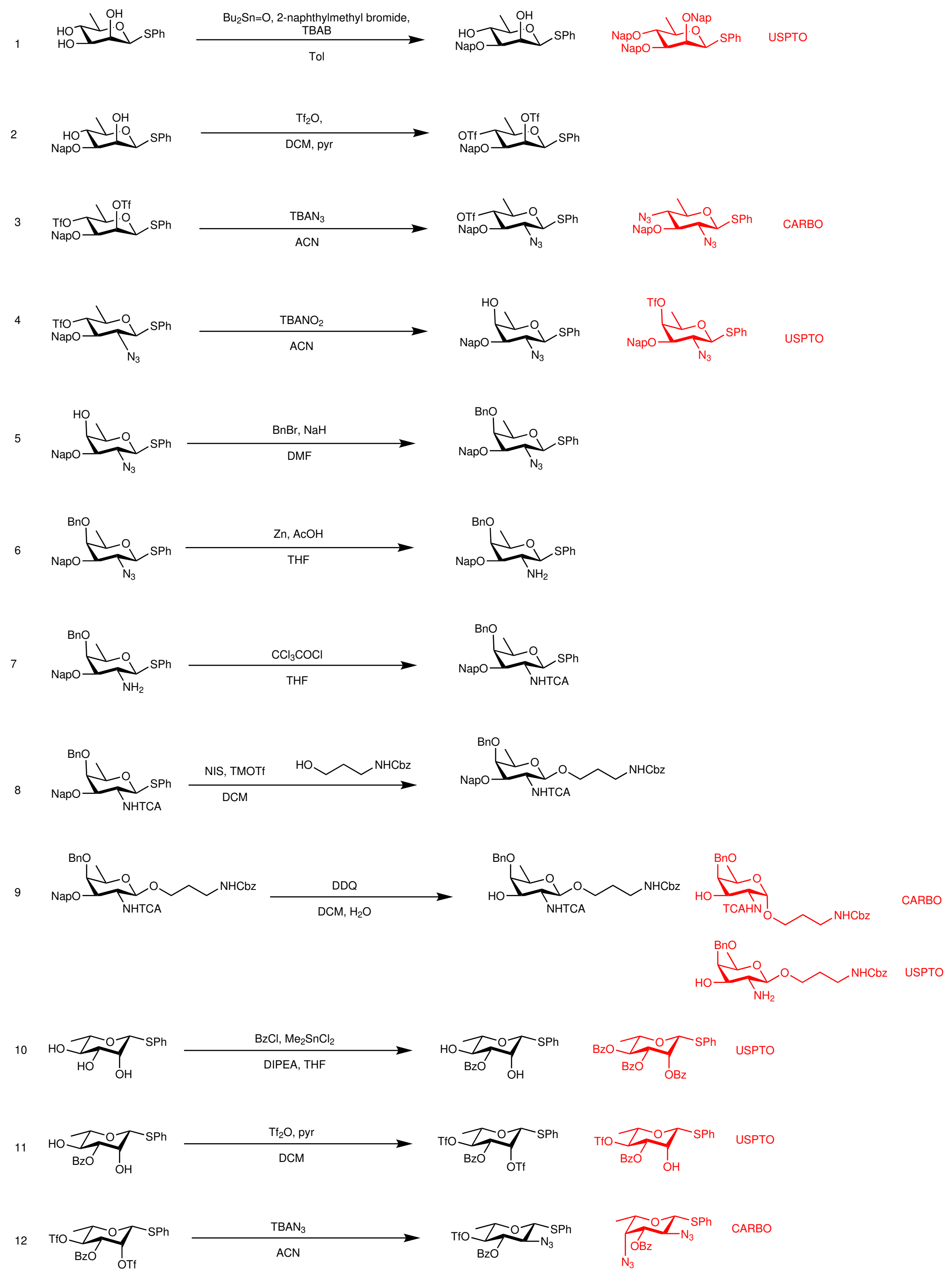

Supplementary Figure 2: Reactions extracted from recent publication[12]. In red structures of wrong prediction by USPTO or CARBO model

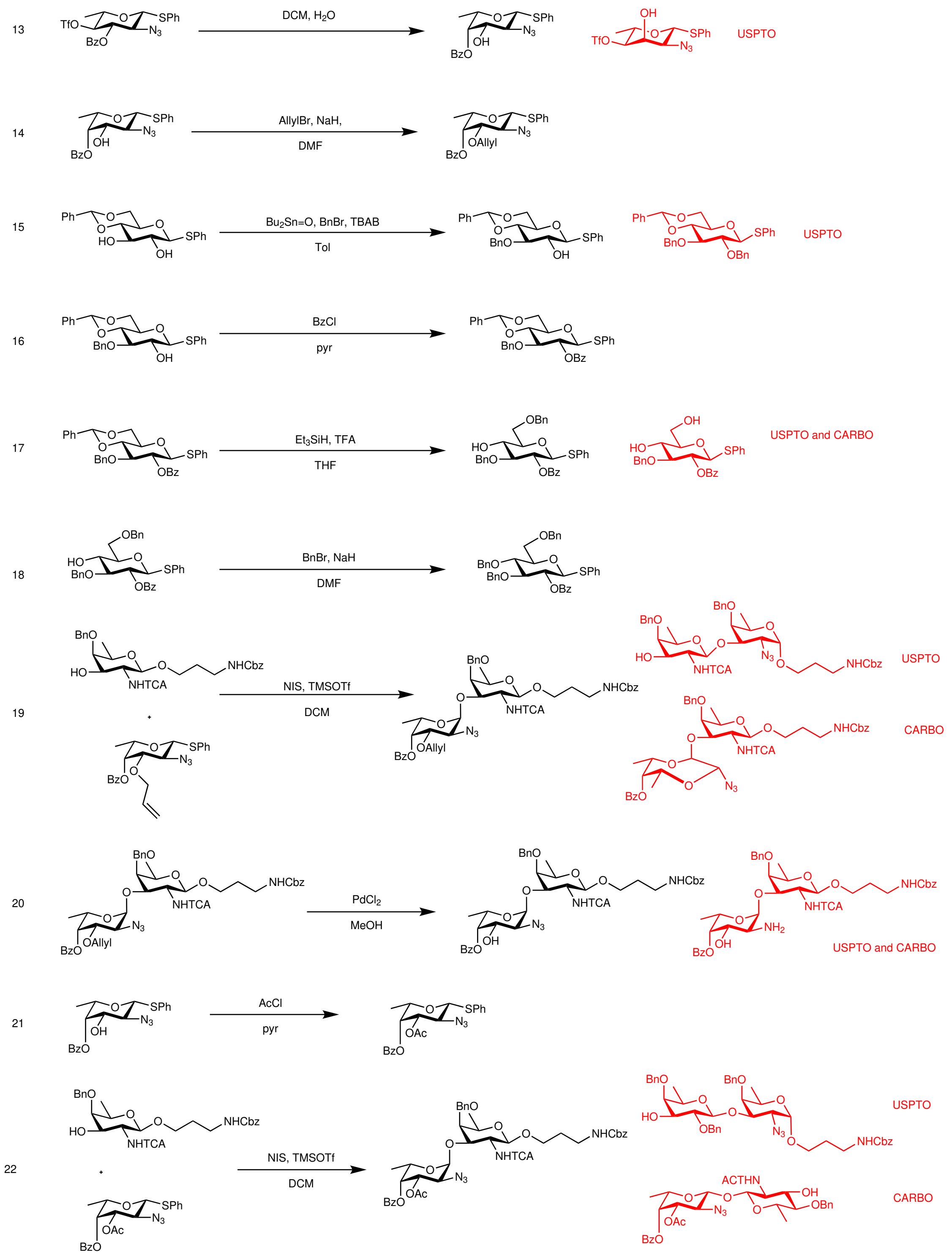

Supplementary Figure 3: Reactions extracted from recent publication[12]. In red structures of wrong prediction by USPTO or CARBO model

23

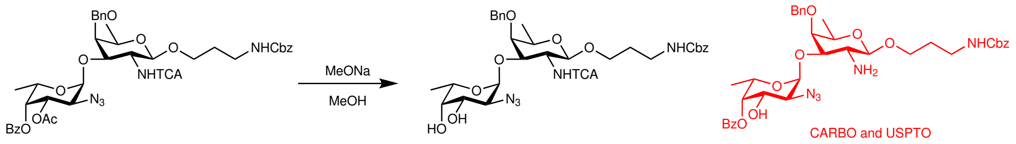

24

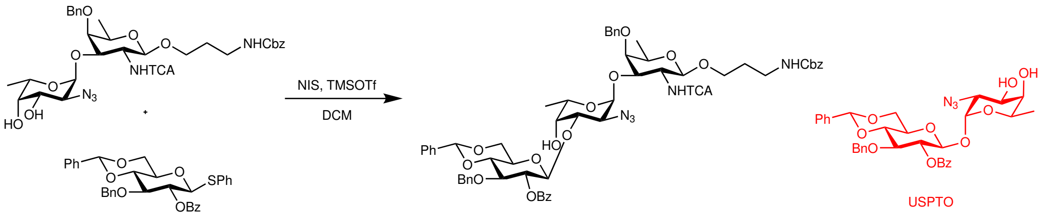

25

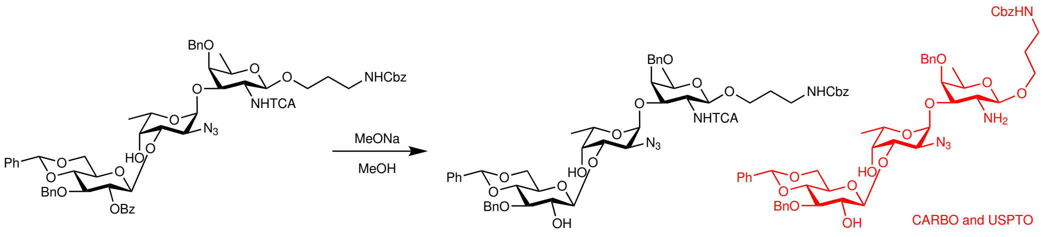

26

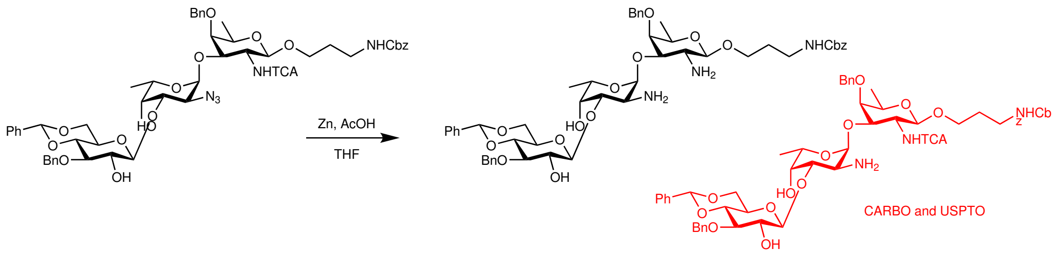

27

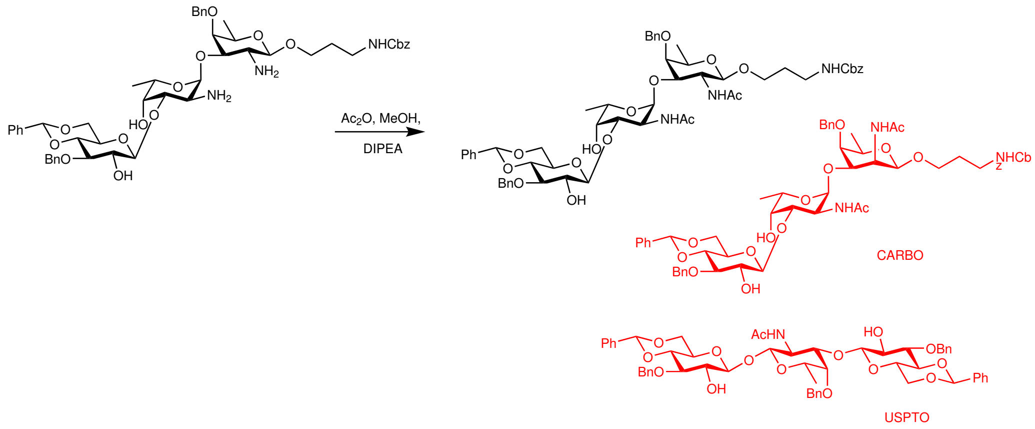

Supplementary Figure 4: Reactions extracted from recent publication[12]. In red structures of wrong prediction by USPTO or CARBO model

28

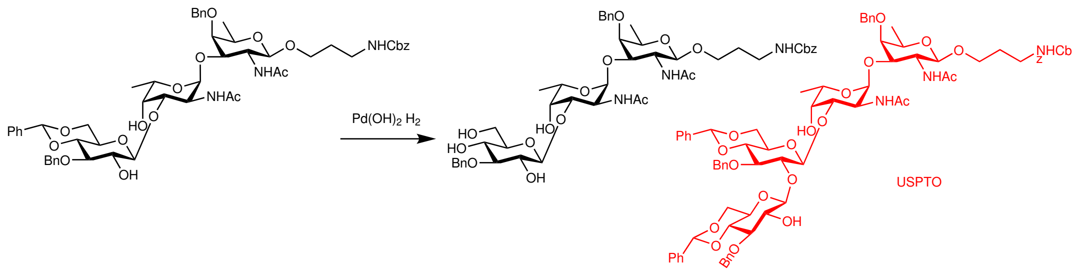

29

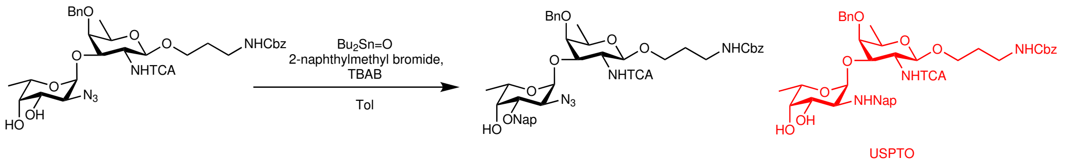

30

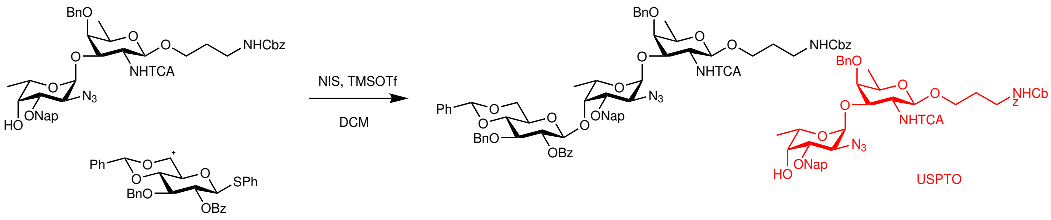

31

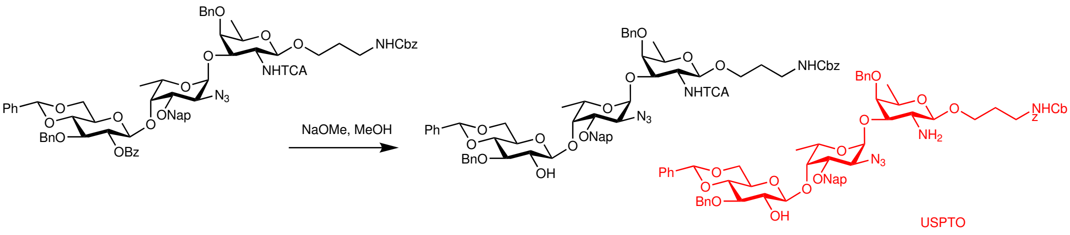

32

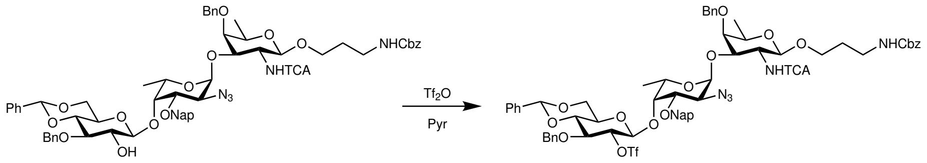

33

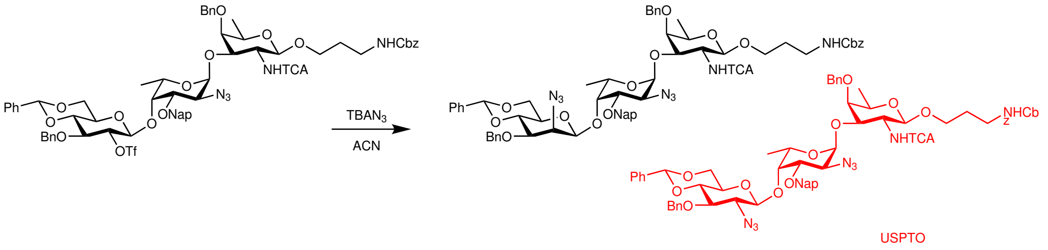

Supplementary Figure 5: Reactions extracted from recent publication[12]. In red structures of wrong prediction by USPTO or CARBO model

34

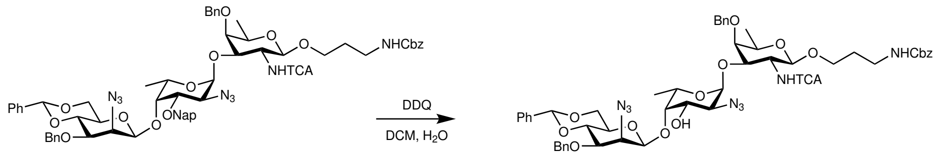

35

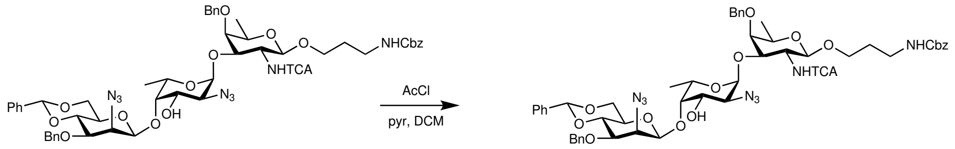

36

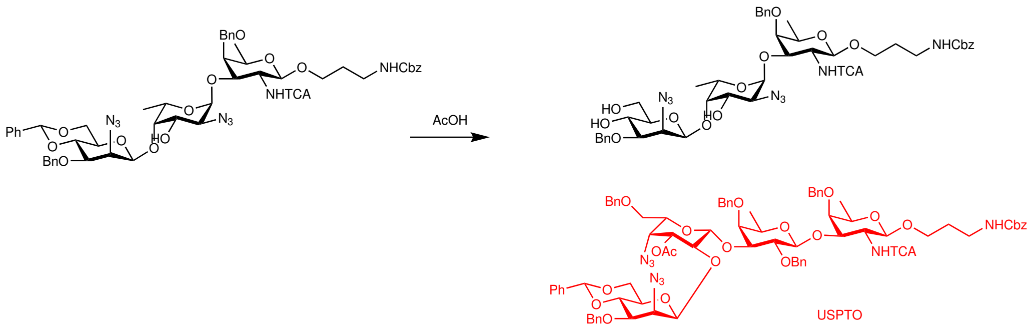

37

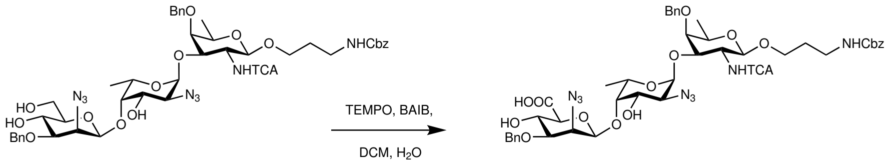

38

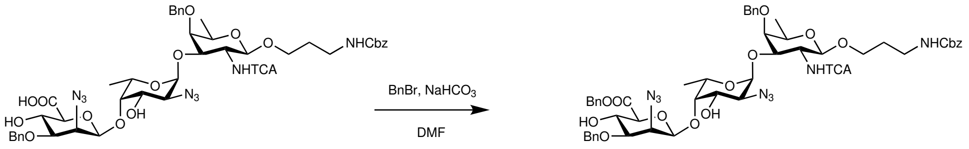

39

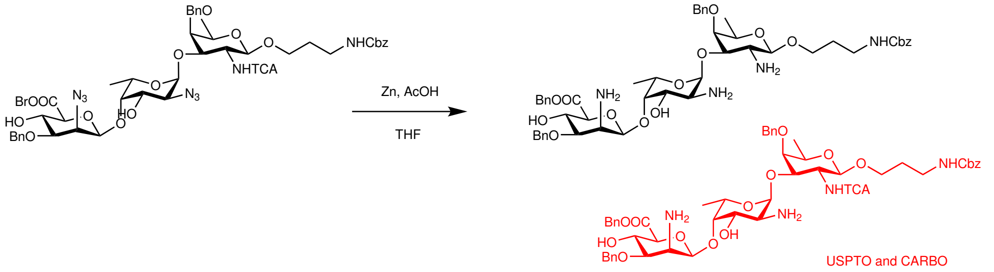

Supplementary Figure 6: Reactions extracted from recent publication[12]. In red structures of wrong prediction by USPTO or CARBO model

Supplementary Table 2: Reactions extracted from recent publication.[12] ([a]): reaction numbering. ([b]): reaction description. ([c]): description of predicted product by USPTO model. ([d]): confidence score of the prediction. ([d]): description of predicted product by CARBO model. Reaction marked with (*) are present in the CARBO data set thus removed from the accuracy calculation.

Rxn[a]	*Reaction*[b]	*USPTO*[c]	*Score*[d]	*CARBO*[c]	*Score*[d]
1	regioselective protection	Peracylation	0.49	correct	0.88
2	Bistriflation	correct	0.78	correct	0.92
3	regioselective substitution	correct	0.89	double substitution	0.99
4	epimerization	inversion of triflate	0.29	correct	1
5	benzylation	correct	0.97	correct	1
6	azide reduction	correct	0.99	correct	0.98
7	TCA formation	correct	0.99	correct	1
8	glycosilation	correct	0.63	correct	0.74
9	Nap ether reduction	TCA deprotection	0.69	epimerization	0.38
10	regioselective benzoylation	perbenzoylation	0.76	correct	0.44
11	bistriflation*	monotriflation*	0.65	correct*	0.89
12	regioselective displacement	correct	0.9	double substitution	1
13	benzoyl migration*	debenzoylation*	0.38	correct*	0.99
14	allilation	correct	0.55	correct	0.73
15	regioselective protection	perbenzoylation	0.15	correct	0.98
16	benzoylation*	correct*	0.96	correct*	0.86
17	benzylidene reduction	benzylidene hydrolysis	0.46	benzylidene hydrolysis	1
18	benzylation	correct	0.9	correct	1
19	glycosilation	wrong anomer	0.09	alchemy	0.2
20	allyl deprotection	azide reduction	0.09	azide reduction	0.53
21	benzoylation	correct	0.64	correct	0.71
22	glycosilation	wrong	0.14	wrong	0.18
23	ester deprotection	TCA deprotection	0.2	TCA dep.	0.38
24	regiosel. glycosilation	non sense	0.22	correct	0.77
25	ester deprotection	TCA deprotection	0.21	TCA deprotection	0.72
26	TCA deprotection	no TCA deprotection	0.71	no TCA deprotection	0.45
27	amine acetylation	non sense	0.12	epimerization	0.36
28	benzylidene reduction	alchemy	0.6	correct	0.88
29	regioselective protection	alchemy	0.42	correct	1

30	glycosilation	no reaction	0.1	correct	0.98
31	ester deprotection	TCA deprotection	0.59	correct	0.62
32	trflation	correct	0.9	correct	1
33	displacement	wrong stereo	0.29	correct	0.77
34	Nap ether reduction	correct	0.37	correct	0.88
35	acetylation	alchemy	0.04	correct	0.4
36	benzylidene hydrolisis	no reaction	0.52	correct	0.99
37	TEMPO oxidation	correct	0.99	correct	0.98
38	benzylation	correct	0.8	correct	1
39	reduction TCA + N_3	no reduction tca	0.64	no reduction tca	0.39
40	acetylation	incomplete acylation	0.23	correct	0.98
41	reduction	correct	0.77	correct	0.91

Supplementary Note 3: Chemical synthesis

General informations. Abbreviations: FCC = Flash Column Chromatography. EtOAc = Ethyl Acetate. DCM = Dichloromethante. Hex = Hexanes. DMF = dimethyl formammide. THF = tetrahydrofuran. MeOH = methanol. DIPEA = N,N-Diisopropylethylamine.

Benzyl-2-acetamido-3,4,6-tri-O-acetyl-2-deoxy-β-D-glucopyranoside (2)

2-acetamido-1,3,4,6-tetra-O-acetyl-2-deoxy-β-D-glucopyranose **1** (9.5 g, 24 mmol) and $Yb(OTf)_3$ (1.49 g, 2.4 mmol) were dissolved in dry and degassed dichloroethane (200 ml). To the mixture benzyl alcohol (7.6 ml 73.2 mol) was added and stirred 2 h at 90° C. After cooling down, the mixture was diluted with DCM (90 ml) and washed with saturated $NaHCO_3$ solution, dried over sodium sulfate and evaporated under reduced pressure. The residue was recrystallized from MeOH/Hexanes to afford **2** (8.5 g 19.4 mmol, 81%) as white crystals. **^{1}H NMR** (400 MHz, $CDCl_3$) δ = 7.31 (bs, 5H, Ar-H), 5.79-5.76 (m, 1H), 5.26-5.19 (m, 1H), 5.11-5.05 (m, 1H), 4.96-4.87 (m, 1H), 4.68-4.58 (m, 1H), 4.29-4.14 (m, 1H), 4.03-3.94

40

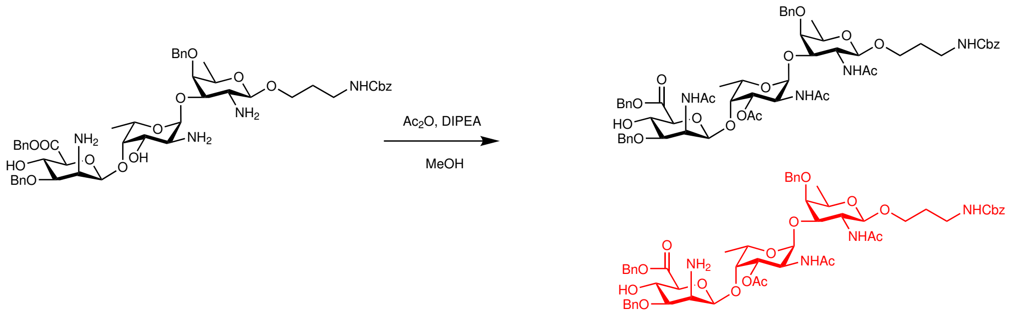

41

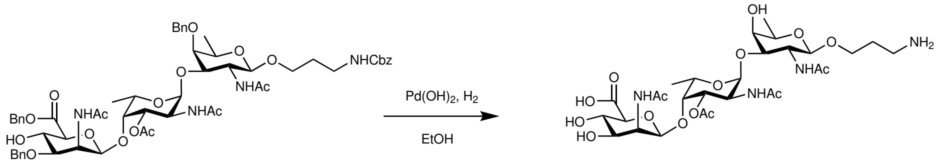

Supplementary Figure 7: Reactions extracted from recent publication[12]. In red structures of wrong prediction by USPTO or CARBO model

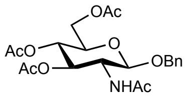

(m, 1H), 3.70-3.67 (m, 1H), 2.10 (s, 3H), 2.01 (s, 6H), 1.88 (s, 3H). Spectroscopic data in agreement with literature.[13]

6-deoxy-6-iodo-benzyl-2-acetamido-2-deoxy-β-D-glucopyranoside (4)

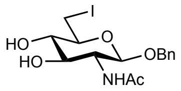

To a stirred solution of **2** (6.12 g, 14 mmol) in 140 mL of MeOH, 280 μL of NaOMe solution 25% were added and the mixture was sonicated for 30'. Upon disappearance of starting material the pH of the mixture was adjusted to 7 with amberlyst® 15 hydrogen form (sigma-aldrich). The resin was filtered, and the organic phase evaporated under reduced pressure. A solution of I_2 (5.44 g, 21.45 mmol) in 25 mL of THF was added to a refluxing suspension of the triol, imidazole (1.95 g, 28.6 mmol) and triphenyl phosphine (5.6 g, 21.4 mmol) in 160 mL of THF. The mixture was refluxed for 30 min, cooled to room temperature and the excess of iodine was quenched with 25 mL of saturated $Na_2S_2O_3$ solution. The

mixture is diluted with 1L of AcOEt and washed with brine. Upon concentration to around one half of the volume the product precipitated as crystalline solid (5.32 g, 12.6 mmol, 88%). **^{1}H NMR** (400 MHz, DMSO-d$_6$) δ = 7.84-7.79 (m, 1H), 7.34-7.27 (m, 5H, Ar-H), 5.39 (bs, 1H), 5.13-5.09 (m, 1H), 4.77 (d, J = 12.51 Hz, 1H), 4.54 (d, J = 12.50 Hz, 1H), 4.45 (d, J = 8.34 Hz, 1H), 3.59-3.47 (m, 2H), 3.03-3.01 (m, 2H), 1.81 (s, 3H). **^{13}C NMR** (101 MHz, DMSO-d$_6$) δ = 169.1, 137.9, 128.2, 127.4, 127.3, 127.3, 127.2, 100.4, 74.6, 74.3, 73.4, 69.6, 55.3, 23.0, 9.2. **ESI-MS (+)** *m/z* calc 444.0284 for $C_{15}H_{20}INO_5Na^+$ (M + Na$^+$), found 444.0260.

Benzyl-2-acetamido-2,6-dideoxy-β-D-glucopyranoside (5)

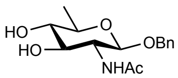

To a suspension of **4** (4 g, 9.5 mmol) and Pd/C (10 % w/w) in 140 mL of THF/Water : 4/1, 1.3 mL of NH_4OH solution 20 % were added. The flask was then charged with H_2 and stirred vigorously for 30'. The mixture was filtered over a pad of celite which was washed with methanol. The residue was concentrated to dryness *in vacuo.* The resulting brown residue was dissolved in a minimum amount of *i*PrOH and the product was precipitated by adding cold diethyl ether as a white solid. (2.2 g, 7.4 mmol, 77%). **^{1}H NMR** (400 MHz, MeOD-d$_4$) δ = 7.33-7.27 (m, 5H, Ar-H), 4.83 (d, 1H, J = 12.94 Hz), 4.57 (d, 1H, J = 12.94 Hz), 4.48 (d, J = 8.53 Hz, H-1), 3.76 (dd, J^1 = 8.5 Hz, J^2 = 10.27 Hz, 1H, H-2), 3.40 (dd, J^1 = 8.89 Hz, J^2 = 10.24 Hz, 1H, H-3), 3.39-3.33 (m, 1H, H-5), 3.06 (m, H1, H-4), 1.99 (s, 3H, NHC=OCH$_3$), 1.33 (d, J = 6.14 Hz, 3H, H-6). **^{13}C NMR** (101 MHz, MeOD-d$_4$) δ = 174.0, 139.0, 129.3, 128.9, 128.8, 101.7, 77.5, 75.8, 73.3, 72.0, 57.4, 23.0, 18.1. **ESI-MS (+)** *m/z* calc 296.1492 $C_{15}H_{22}NO_5$ (m + H$^+$), found 296.1481.

3-Benzoyl-benzyl-2-acetamido-2,6-dideoxy-β-D-glucopyranoside (6)

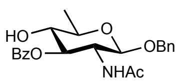

A solution of **5** (1.46 g, 4.94 mmol) in 50 mL of dry pyridine was cooled at -38° C using a cryostat and benzoylchloride (630 uL, 5.43 mmol) was added slowly dropwise under efficient stirring. After the addition was completed the mixture was stirred 1.5 h at the same temperature and then quenched with methanol. The mixture was diluted in 700 mL of EtOAc, washed with 0.1 M HCl, water and $NaHCO_3$ sequentially. The organic phase was then dried with sodium sulfate, filtered and evaporated under reduced pressure. The residue was dissolved in 20 mL of DCM and the product was precipitated by adding 80 mL of cold hexane as a white solid (1.06 g, 2.65 mmol 53%). The solid was filtered and the mother liquor was evaporated. The residue was purified by FCC on silica gel (Hex/EtOAc : 1/1) that gave **6** as a white solid (322 mg, 0.81 mmol, 17% . tot yield: 70%). **^{1}H NMR** (400 MHz, MeOD-d$_4$) δ = 8.03-8.01 (m, 2H, Ar-H), 7.61-7.57 (m, 1H, Ar-H), 7.48-7.44 (m, 2H, Ar-H), 7.33-7.26 (m, 5H, Ar-H), 5.23-5.18 (dd, J^1 = 9.10 Hz, J^2 = 10.55 Hz, 1H, H-3), 4.66 (d, J = 8.54Hz, 1H, H-1), 4.60 (d, J = 12.04 Hz, 1H), 4.05 (dd, J^1 = 8.40 Hz, J^2 = 10.53 Hz, 1H, H-2), 3.51-3.57 (m, 1H, H-5), 3.42-3.35 (m, 1H, H-4), 1.78 (s, 3H, NHC=OC$\underline{H_3}$), 1.38 (d, J = 6.07 Hz, 3H, H-6).**^{13}C NMR** (101 MHz, MeOD-d$_4$) δ = 173.3, 167.9, 139.0, 134.3, 131.4, 130.8, 129.5, 129.3, 128.8, 128.7, 101.5, 77.4, 75.3, 73.4, 71.9, 55.7, 22.7, 18.04.

Benzyl 3,4,6-tri-O-acetyl-2-deoxy-2-[(2,2,2-trichloroethoxy)carbonyl]-amino-D-glucopyranosyl-β-(1→4)-2-acetamido-3-O-benzoyl-2,6-dideoxy-β-D-glucopyranoside (8)

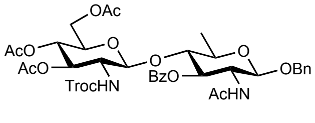

To a solution of **6** (964 mg, 2.37 mmol) and **7**[14] (2.96 g, 4.74 mmol) in 80 mL of DCM, freshly activated powdered 4 Å molecular sieve were added and the mixture cooled to -20° C.

Boron trifluoride diethyl etherate (525 μL, 4.26 mmol) was added dropwise and the mixture was allowed to warmup to rt overnight and stirred for a total of 28 h. The reaction was quenched with 1 mL of DIPEA, filtered over celite and evaporated. The crude mixture was purified by FCC (hex/EtOAc : 4/6). The collected fractions were concentrated to dryness in vacuo and product was precipitated from AcOEt with cold hexane to yield **8** as a white solid (1.502 g, 1.74 mmol, 73%). **^{1}H NMR** (400 MHz, $CDCl_3$) δ = 8.02-7.99 (m, 2H, Ar-H), 7.56-7.51 (m, 1H, Ar-H), 7.40-7.31 (m, 7H, Ar-H), 5.52 (d, J = 5.52 Hz), 5.41-5.20 (m, 3H), 4.91-4.73 (m, 4H), 4.62-4.48 (m, 3H), 4.26-4.13 (m, 1H), 3.79-3.67 (m, 2H), 3.59-349 (m, 3H), 3.35-3.32 (m, 1H), 1.96, 1.94, 1.92, (3x s, 3H, OC=OCH$_3$) 1.81, (s, 3H, NHC=OC$\underline{H_3}$) 1.40 (d, J = 6.40 Hz, 3H). **^{13}C NMR** (101 MHz, $CDCl_3$) δ = 170.7, 170.6, 170.4, 169.4, 166.5, 154.3, 137.3, 133.65, 129.9, 129.4, 128.6, 125.55, 128.1, 128.1, 101.0, 99.6, 95.4, 81.8, 74.6, 73.9, 71.9, 71.7, 71.2, 70.5, 68.3, 61.5, 56.9, 54.4, 29.8, 23.3, 20.8, 20.7, 20.6, 18.1. **ESI-MS (+)** *m/z* calc 861.806 for $C_{37}H_{44}Cl_3N_2O_{15}^+$ (M + H$^+$), found 861.1775.

Benzyl 3,4,6-tri-O-acetyl-2-deoxy-2-N-acetoammido-D-glucopyranosyl-β-(1$\rightarrow$ 4)-2-acetamido-3-O-benzoyl-2,6-dideoxy-deoxy-β-D-glucopyranoside (9)

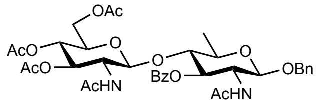

To a solution of **8** (400 mg, 0.46 mmol) in 9 mL of a DCE, acetic acid and acetic anhydride in 4:5:1 mixture, zinc dust (905 mg, 13.9 mmol), was added and the mixture stirred at 60° C in a sealed tube for 3 h. The mixture was concentrated to dryness under reduced pressure and the residue purified by FCC (DCM/Acetone : 7/3) to yield **9** (319 mg, 0.44 mmol, 96 %) as a white powder. **^{1}H NMR** (400 MHz, MeOD-d$_4$) δ = 8.07-8.04 (m, 2H, Ar-H), 7.61-7.59 (m, 1H, Ar-H), 7.49-7.45 (m, 2H, Ar-H), 7.33-7.26 (m, 5H, Ar-H), 5.35-5.30 (m, 1H), 5.15-5.10 (m, 1H), 4.83 (m, 1H), 4.80-4.73 (m, 2H), 4.66-4.61 (m, 2H), 4.10-4.05 (m, 1H), 3.74-3.55 (m, 4H), 3.39 (dd, J^1 = 12.34 Hz, J^2 = 2.54 Hz, 1H), 1.94, 1.91, 1.88, 1.87 (4 x s, 3H, OC=OCH$_3$), 1.75 (s, 3H, HNC=OC$\underline{H_3}$), 1.38 (d, J = 6.05 Hz,

3H). **^{13}C NMR** (101 MHz, MeOD-d$_4$) δ = 173.4, 173.3, 172.1, 171.8, 171.1, 171.1, 167.0, 138.9, 134.5, 131.3, 130.8, 129.7, 129.4, 128.8, 102.6, 101.2, 83.5, 75.8, 73.8, 72.6, 72.2, 71.9, 69.7, 62.6, 56.00, 55.9, 22.9, 22.6, 20.7, 20.5, 20.5, 18.2. **ESI-MS (+)** *m/z* calc 728.2793 for $C_{36}H_{44}N_2O_{14}Na^+$ (M+Na$^+$), found 751.2685.

Benzyl 3,4,6-tri-O-acetyl-2-deoxy-2-N-acetoammido-D-glucopyranosyl-β-(1→4)-2-acetamido-3-O-benzoyl-2,6-dideoxy-β -D-glucopyranoside (10)

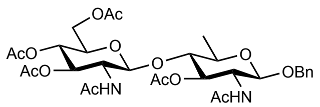

To a solution of **9** (353 mg, 0,48 mmol) in 8 mL of a mixture MeOH/DMF : 4/1, sodium methoxide (120 μl of a solution 25% in methanol) was added and the mixture stirred for 4 days. The reaction was diluted with 1 mL of water and the pH was adjusted to 7 with Amberlyst 15 hydrogen form. The mixture was filtered and concentrated to dryness under reduced pressure. The residue was dissolved in 4 mL of dry pyridine and acetic anhydride (390 uL, 3.84 mmol) and 4-(Dimethylamino)pyridine (5 mg, 0.04 mmol) were sequentially added at rt. After 1 h the reaction was quenched with methanol and concentrated to dryness. The residue was purified by FCC on silica gel (DCM/Acetone : 7/3) to afford **10** as a white powder (255 mg, 3.89 mmol, 80%). **^{1}H NMR** (400 MHz, DMSO-d$_6$) δ = 8.05 (d, J = 9.25 Hz, 1H, N$\underline{H}$Ac), 7.90 (d, J = 9.25 Hz, 1H, N$\underline{H}$Ac), 7.35-7.26 (m, 5H, Ar-H), 5.14, 5.09 (m, 1H,) 4.92-4.87 (m, 1H), 4.84-4.79 (m, 1H), 4.75-4.70 (m, 1H), 4.85 (d, J = 8.85 Hz, 1H), 4.50 (d, J = 12.06 Hz, 1H), 4.27 (dd, J^1 = 12.53 Hz, J^2 = 4.02 Hz), 3.90-3.81 (m, 2H), 3.76-3.69 (m, 1H), 3.63-3.56 (m, 1H), 3.43-3.39 (m, 1H), 2.00, 1.95, 1.94, 1.90, (4 x s, 3H, OC=OC$\underline{H_3}$), 1.76, 1.74 (2 x s, 3H, NHC=OCH$_3$), 1.24 (d, J = 5.23 Hz, 3H). **^{13}C NMR** (101 MHz, DMSO-d$_6$) δ = 170.0, 169.6, 169.4, 169.2, 169.2, 169.0, 137.8, 128.1, 127.4, 127.2, 100.6, 100.0, 80.9, 73.5, 72.4, 70.4, 70.3, 70.1, 68.3, 61.6, 53.7, 53.6, 22.7, 22.6, 20.4, 20.4, 20.4, 20.3, 17.4. **ESI-MS (+)** *m/z* calc 689.2534 for $C_{31}H_{42}N_2NaO_{14}^+$ (m + H$^+$), found 689.2520.

3,4,6-tri-O-acetyl-2-deoxy-2-N-acetoammido-D-glucopyranosyl-β-(1$\rightarrow$ 4)-2-acetamido-3-O-acetyl-2,6-dideoxy-α-D-glucopyranoside-1-dibenzyl phosphate (12)

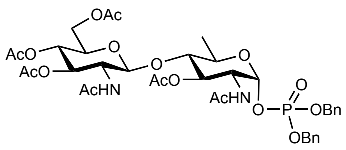

To a solution of **10** (62 mg, 93 μmol) in 3 mL of THF/MeOH : 4/1 palladium on activated charcoal was added (10% w/w) and the flask was charged with a hydrogen atmosphere. The mixture was stirred vigorously for 16 h and then filtered over celite and concentrated to dryness. The residue was dissolved in 3 mL of a mixture THF/DMF : 2/1 and cooled to -78° C. Lithium bis(trimethylsilyl)amide 1 M solution in THF (96 μL, 96 μmol) was added slowly dropwise and stirred for 15' at the same temperature. Tetrabenzyl pyrophosphate (51 mg, 96 μmol) was dissolved in 200 μL of THF and added to the reaction mixture, and the containing vial was washed with 100 μL of THF. The mixture was allowed to warm up to rt over ca 4 h. The mixture was then diluted with 20 mL of chloroform and washed once with $NaHCO_3$. The organic phase was dried over sodium sulfate and concentrated to ca 3 mL under reduced pressure. Cold hexane was added (5 mL) and the mixture is cooled down to 0° C until a white precipitate was formed (42 mg, 50 μ mol, 56 %). **^{1}H NMR** (400 MHz, $CDCl_3$) δ = 7.36-7.34 (m, 10H, Ar-H), 5.88-5.86 (m, 1H), 5.75-5.72 (m, 1H), 5.59 (bs, 1H), 5.12-4.99 (m, 6H), 4.88-4.86 (m, 1H), 4.40-4.36 (m, 1H), 4.28-4.23 (m, 1H), 4.00-3.90 (m, 2H), 3.70-3.68 (m, 1H), 3.59-3.53 (m, 1H), 3.48-3.43 (m, 1H), 2.06, 20.2, 2.1, 2.00, 1.89, 1.69 (6 x s, 3H), 1.16 (d, J = 6.00 Hz, 3H). **^{13}C NMR** (101 MHz, $CDCl_3$) δ = 171.3, 170.8, 170.7, 170.5, 169.6, 135.6, 135.5, 135.4, 129.0, 128.9, 128.9, 128.9, 128.5, 128.2, 128.1, 100.4, 96.4 (d, J = 6.41 Hz), 80.6, 71.9, 71.8, 70.4, 69.9 69,9, 69.8, 69.8, 68.5, 61.9, 56.0, 52.3, 52.2, 23.4, 22.9, 20.9, 20.8, 20.7, 17.4. **^{31}P NMR** (122 MHz, $CDCl_3$) δ = -2.29. **ESI-MS (+)** *m/z* calc 837.2847 for $C_{38}H_{50}N_2O_{17}P^+$ (M + H^+), found 837.2841.

Farnesylcitronellyl-pyrophosphoryl-α-3,4,6-tri-O-acetyl-2-deoxy-2-N-acetoammido-

D-glucopyranosyl-β-(1$\rightarrow$ 4)-3-O-acetyl-2-acetamido-2,6-dideoxy-α-D-glucopyranoside (14)

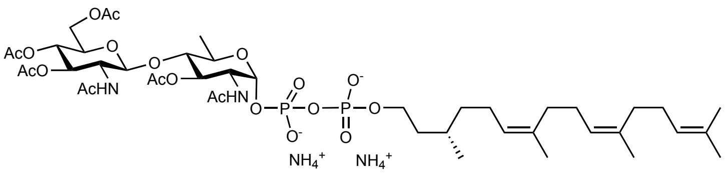

A solution of **12** (62 mg, 93 μmol) and Pd/C (10% w/w) in 3 mL of THF/MeOH : 4/1 was stirred for 16 h under H_2 atmosphere. The mixture was filtered over celite and concentrated to dryness under reduced pressure. The residue was co-evaporated with dry toluene three times, dissolved in 1 mL of DMF and added to the farnesylcitronellyl-phosphoroimidazolidate.[15] The mixture was stirred for 5 days and concentrated to dryness. The crude was purified by FCC (EtOAc/*i*PrOH/H_2O : 4/2/1) using silica gel pretreated with an excess of ammonium hydroxide (25% in water solution) and washed with the eluent. Fractions containing desired product were pooled and lyophilized. The product was further purified by reverse phase chromatography (Sep-Pak cartridge, C-18, 360mg) as follows. The crude was dissolved in 2 mL of a mixture 1:1 MeOH/Water and loaded on the column pre equilibrated with the same eluent. The column was washed with 15 mL of 1:1 MeOH/water and then 15 mL MeOH. Fractions containing the desired product were pooled and evaporated. (5 mg, 4.7 μ mol, 11%). ^{1}**H NMR** (400 MHz, MeOD-d_4) δ = 5.48-5.47 (m, 1H), 5.31-5.26 (m, 1H), 5.14-5.04 (m, 4H), 5.97-4.92 (m, 2H), 4.77-4.75 (m, 2H), 4.43 (dd, J^1 = 12.49 Hz, J^2 = 4.06 Hz, 1H), 4.21-4.19 (m, 1H), 4.06-3.98 (m, 4H), 3.80-3.76 (m, 1H), 3.71-3.67 (m, 1H), 3.49-3.44 (m, 1H), 2.05-1.91 (m, 13H), 1.68-1.61(m, 12H), 1.44-1.34 (m, 3H), 1.27 (d, J = 6.24 Hz, 3H), 1.19-1.13 (m, 1H), 0.91 (d, J = 6.69 Hz, 3H). ^{13}**C NMR** (101 MHz, MeOD-d_4) δ = 170.9, 170.4, 169.9, 125.4, 134.8, 134.3, 130.9, 125.4, 124.8, 124.0, 100.8, 81.5, 72.5, 71.9, 71.3, 68.5, 67.4, 61.6, 54.8, 37.3, 31.8, 31.5, 29.3, 29.1, 26.3, 26.1, 25.0, 24.6, 22.4, 22.32, 21.5, 19.7, 19.3, 19.2, 19.1, 18.4, 16.7, 16.4. ^{31}**P NMR** (122 MHz, MeOD-d_4) δ = -10.1 (d, J = 20.11 Hz), -13.1 (d, J = 20.50 Hz) **ESI-MS (-)** *m/z* calc 1009.4081 for $C_{44}H_{71}N_2O_{20}P_2^-$ found 1009.4078.

Farnesylcitronellyl-pyrophosphoryl-α-2-deoxy-2-N-acetoammido-D-glucopyranosyl-β(1$\rightarrow$ 4)-2-acetamido-2,6-dideoxy-α-D-glucopyranoside (15)

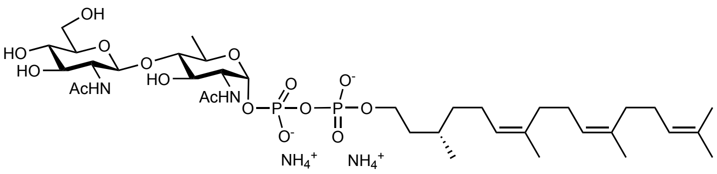

To a stirred solution of **14** (4.8 mg, 4.59 μmol) in 1 mL of MeOH, 0.5 mL of NH_4OH 25% solution in water were added and the mixture was stirred for 16 h and lyophilized to obtain **15** as a white lyophilizate (3.9 mg, quant.). **^{1}H NMR** (400 MHz, MeOD-d_4) δ = 5.47-5.44 (m, 1H), 5.13-5.11 (m, 3H), 4.60-4.58 (m, 1H), 4.07-4.01 (m, 5H), 3.90-3.80 (m, 2H), 3.67-3.50 (m, 4H), 3.22-3.17 (m, 1H), 2.05-1.97 (m, 14H), 1.68-1.62 (m, 15H), 1.41-1.35 (m, 3H), 1.29-1.25 (m, 3H), 1.21-1.15 (m, 1H), 0.92 (d, J = 6.27 Hz, 3H). **^{13}C NMR** (101 MHz, MeOD-d_4) δ = 174.2, 173.5, 136.2, 135.7, 132.3, 126.8, 126.2, 125.4, 103.1, 95.9, 87.7, 78.2, 75.7, 72.0, 71.5, 68.5, 65.6, 62.6, 57.9, 55.0, 38.9, 38.8, 33.22, 32.89, 30.6, 27.7, 27.5, 26.4, 25.9, 23.8, 23.7, 23.1, 23.0, 19.8, 18.1, 17.7. ^{31}P NMR (122 MHz, MeOD-d_4) δ = -10.3 (d, J = 20.1 Hz), -12.9 (d, J = 21.0 Hz). **ESI-MS (-)** m/z calc 841.3658 for $C_{36}H_{63}N_2O_{16}P_2^-$ found 841.3656.

Supplementary Figure 8: Benzyl-2-acetamido-3,4,6-tri-O-acetyl-2-deoxy-β-D-glucopyranoside (2)

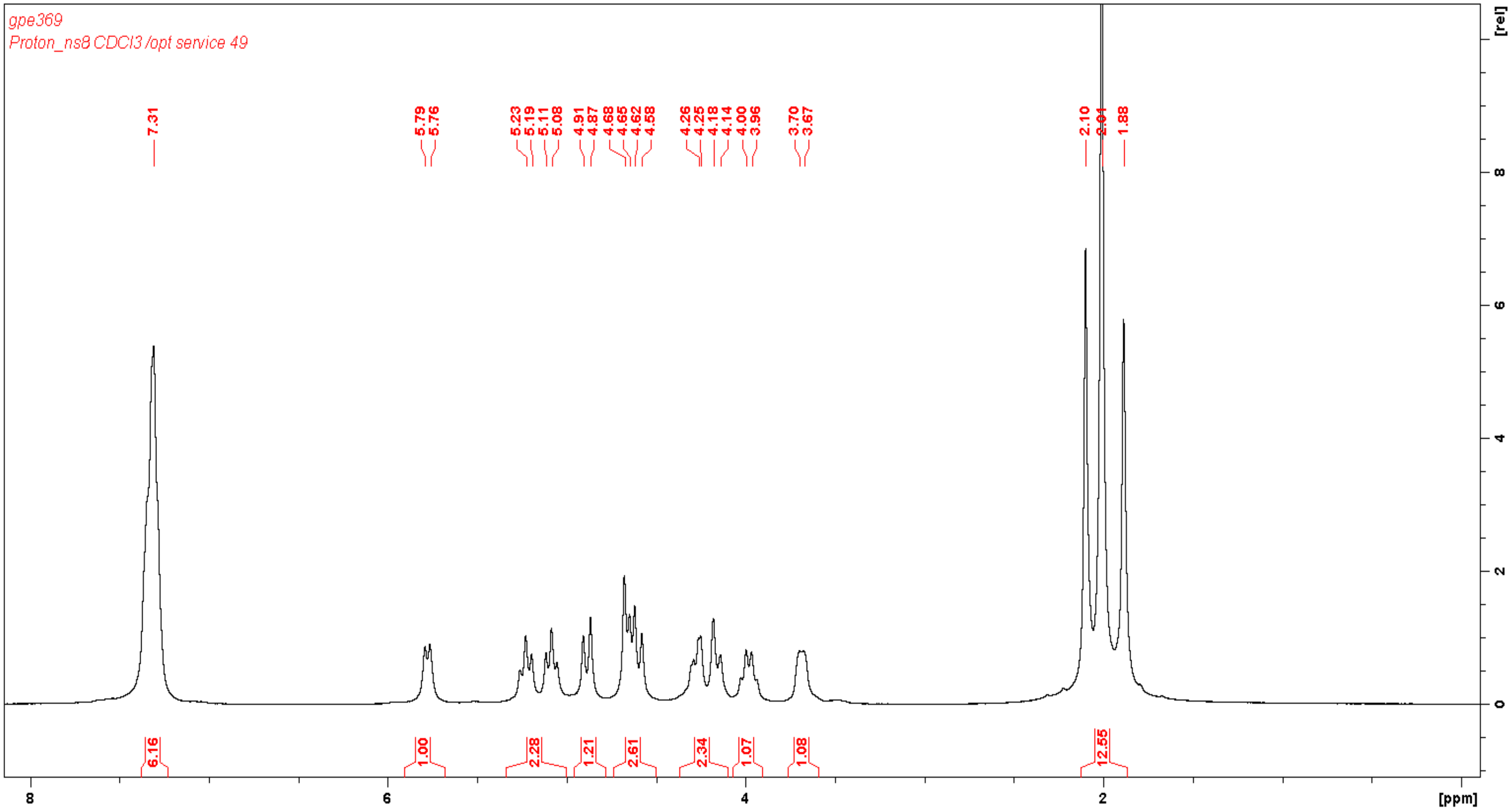

Supplementary Figure 9: 6-deoxy-6-iodo-benzyl-2-acetamido-2-deoxy-β-D-glucopyranoside (4) ^{1}H NMR

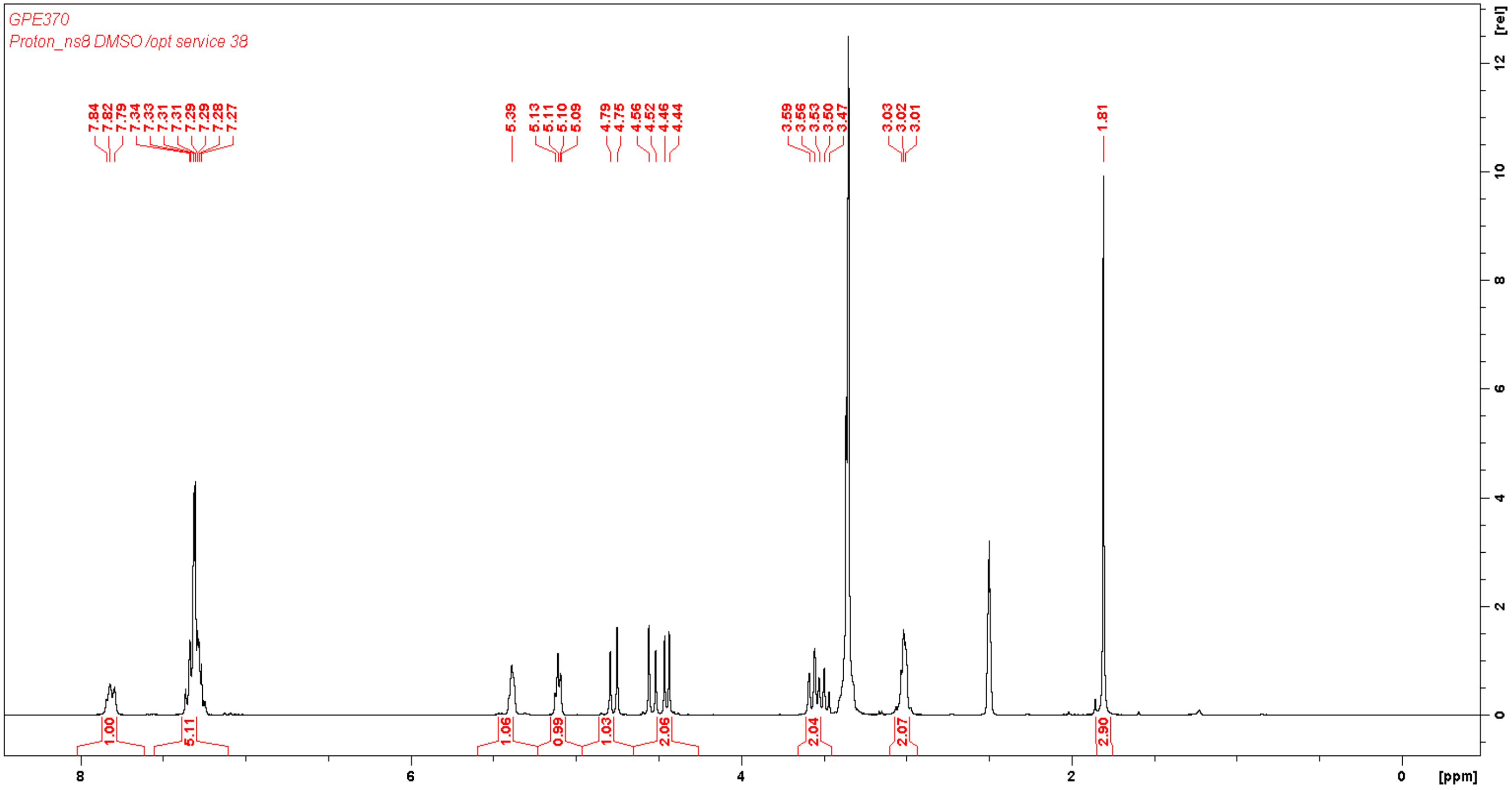

Supplementary Figure 10: 6-deoxy-6-iodo-benzyl-2-acetamido-2-deoxy-β-D-glucopyranoside (4) ^{13}C NMR

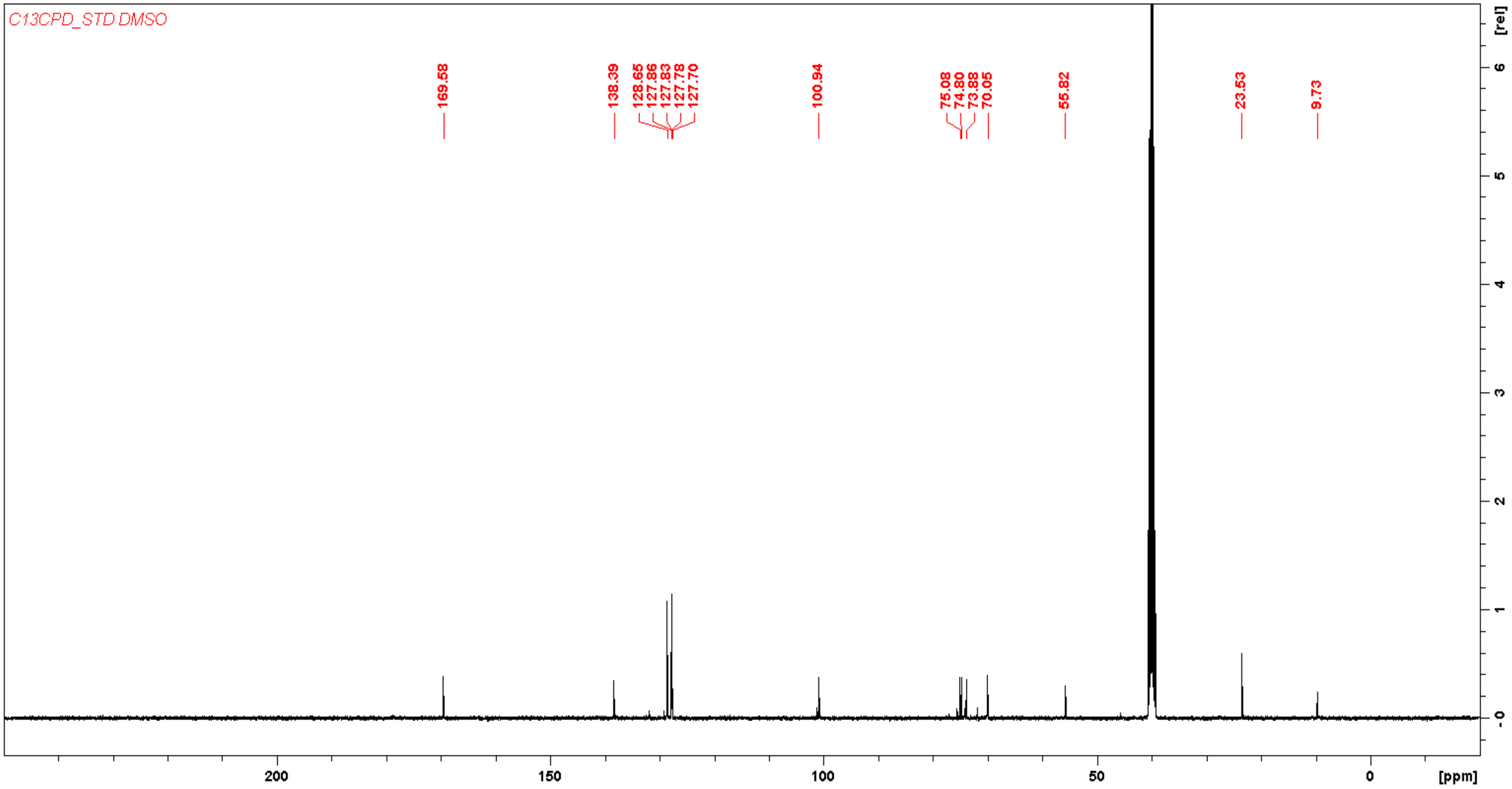

Supplementary Figure 11: benzyl-2-acetamido-2,6-dideoxy-β-D-glucopyranoside (5) $_1$H NMR

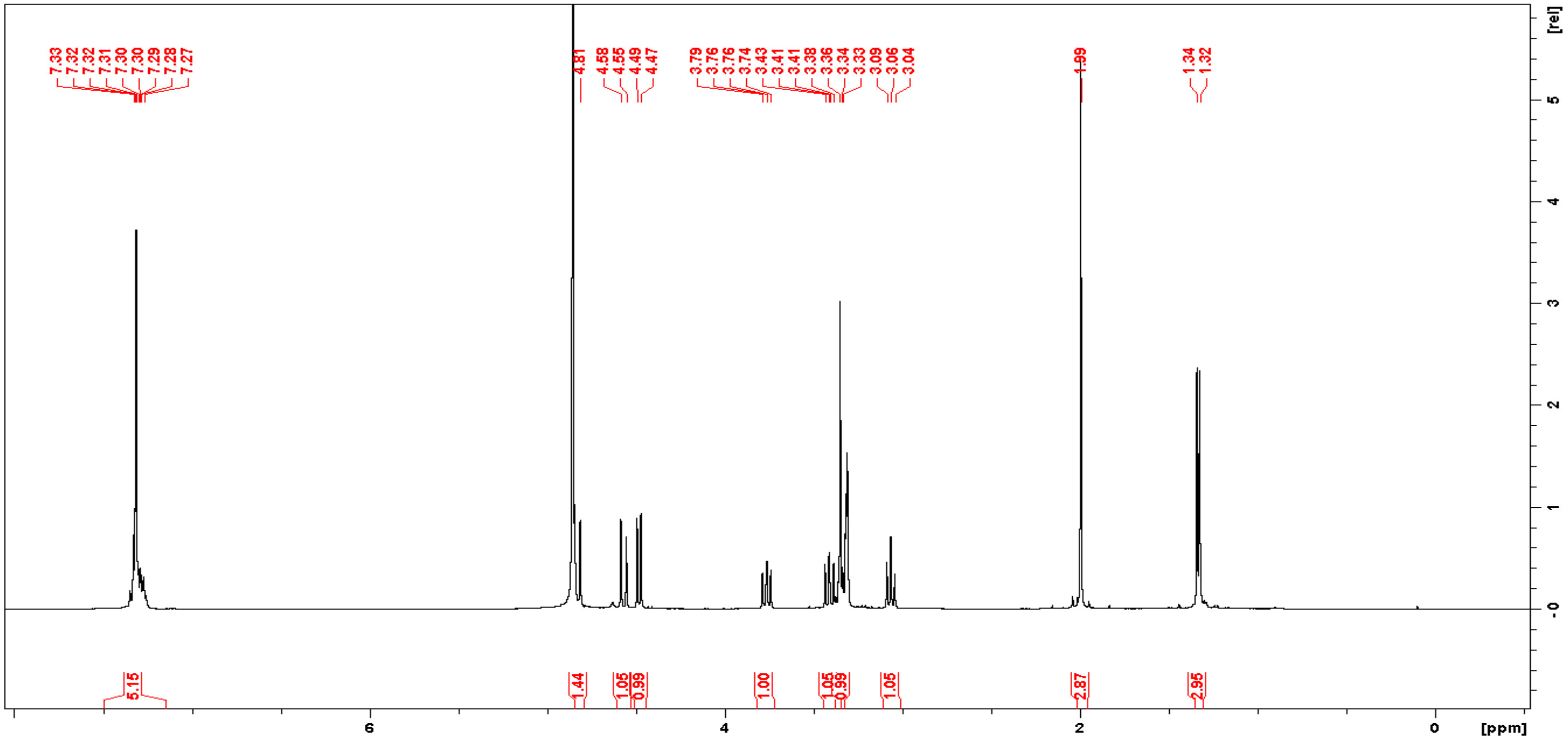

Supplementary Figure 12: benzyl-2-acetamido-2,6-dideoxy-β-D-glucopyranoside (5) ^{13}C NMR

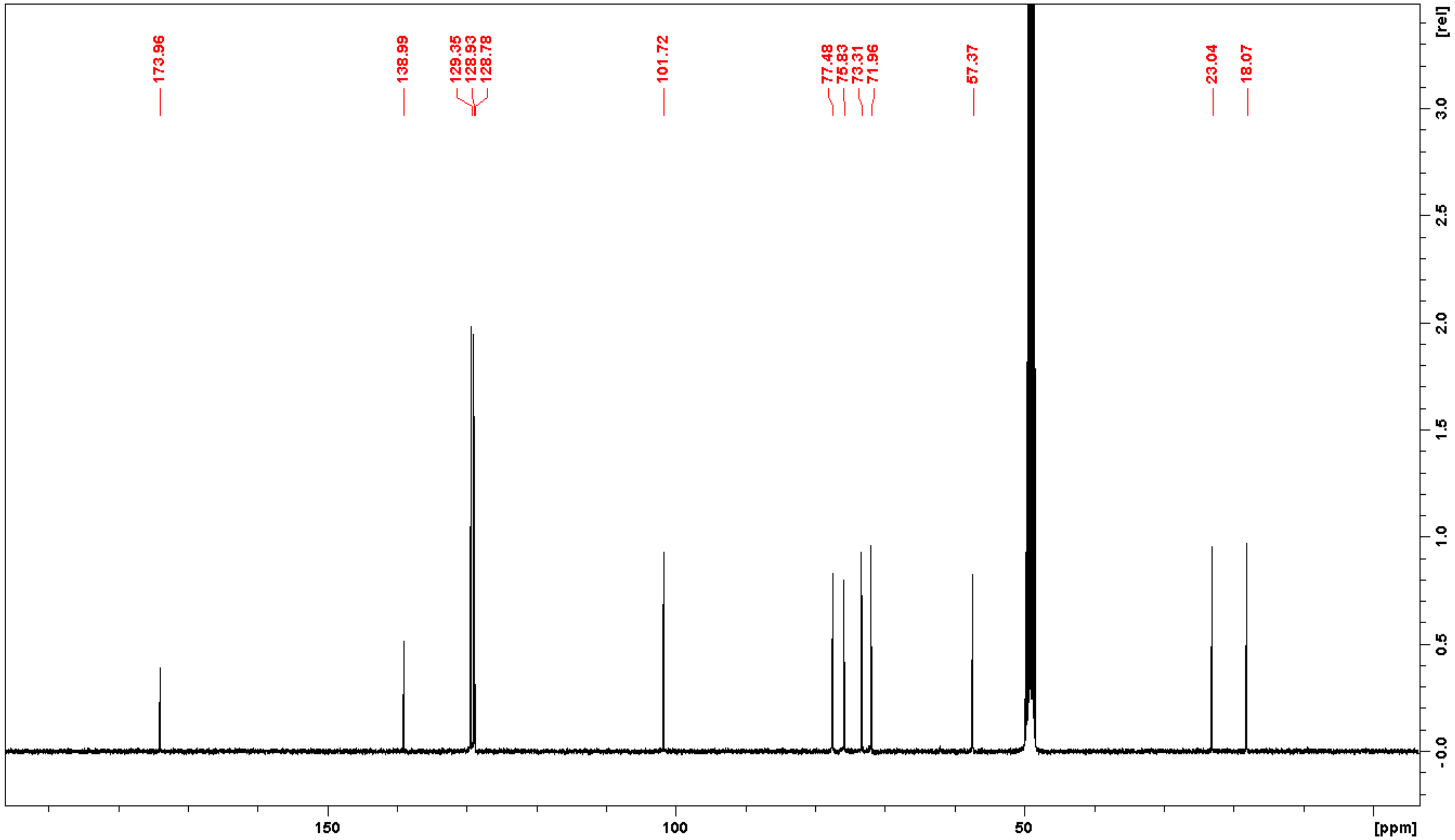

Supplementary Figure 13: 3-Benzoyl-benzyl-2-acetamido-2,6-dideoxy-β-D-glucopyranoside (6) ^{1}H NMR

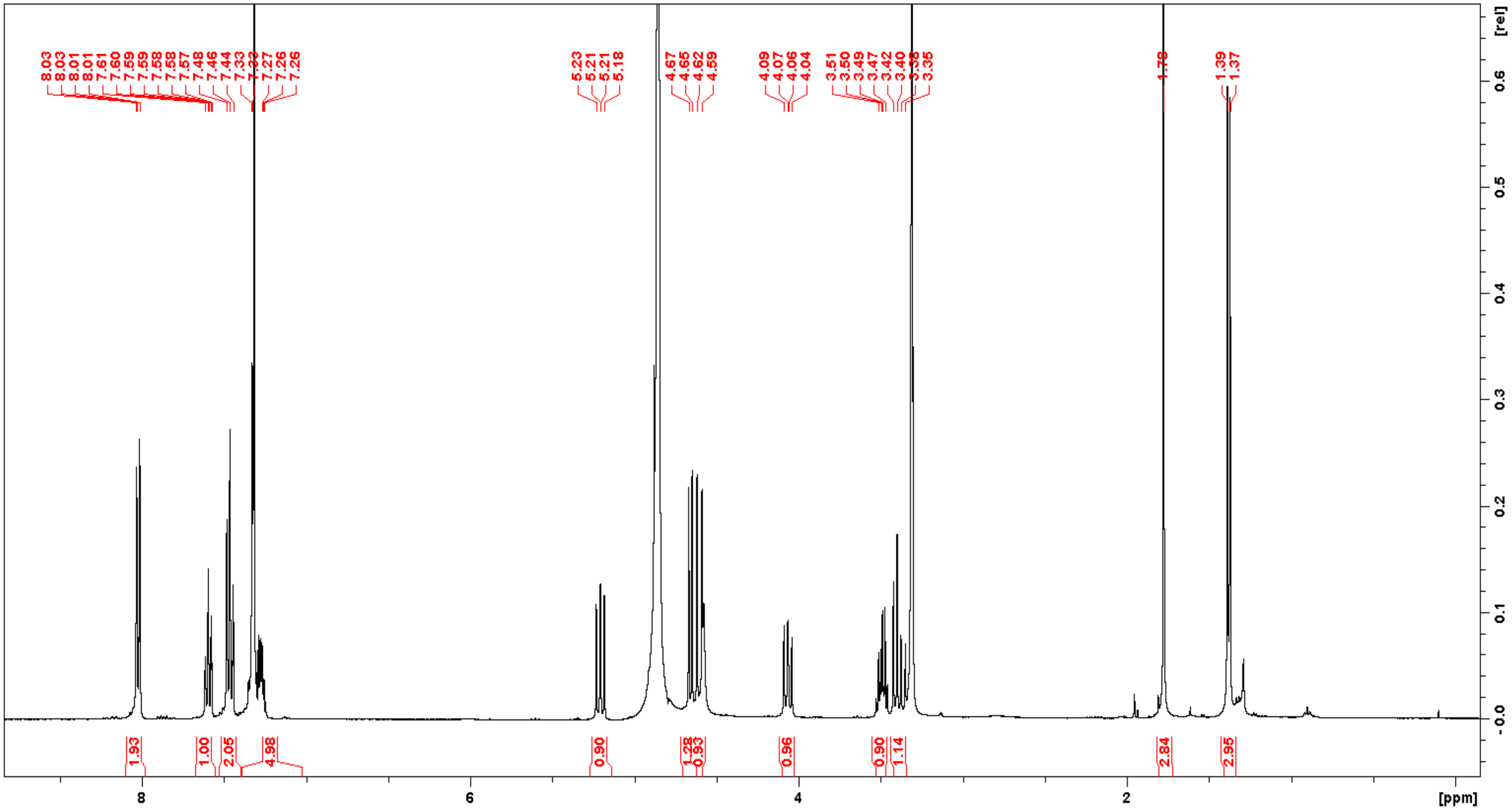

Supplementary Figure 14: 3-Benzoyl-benzyl-2-acetamido-2,6-dideoxy-β-D-glucopyranoside (6) ^{13}C NMR

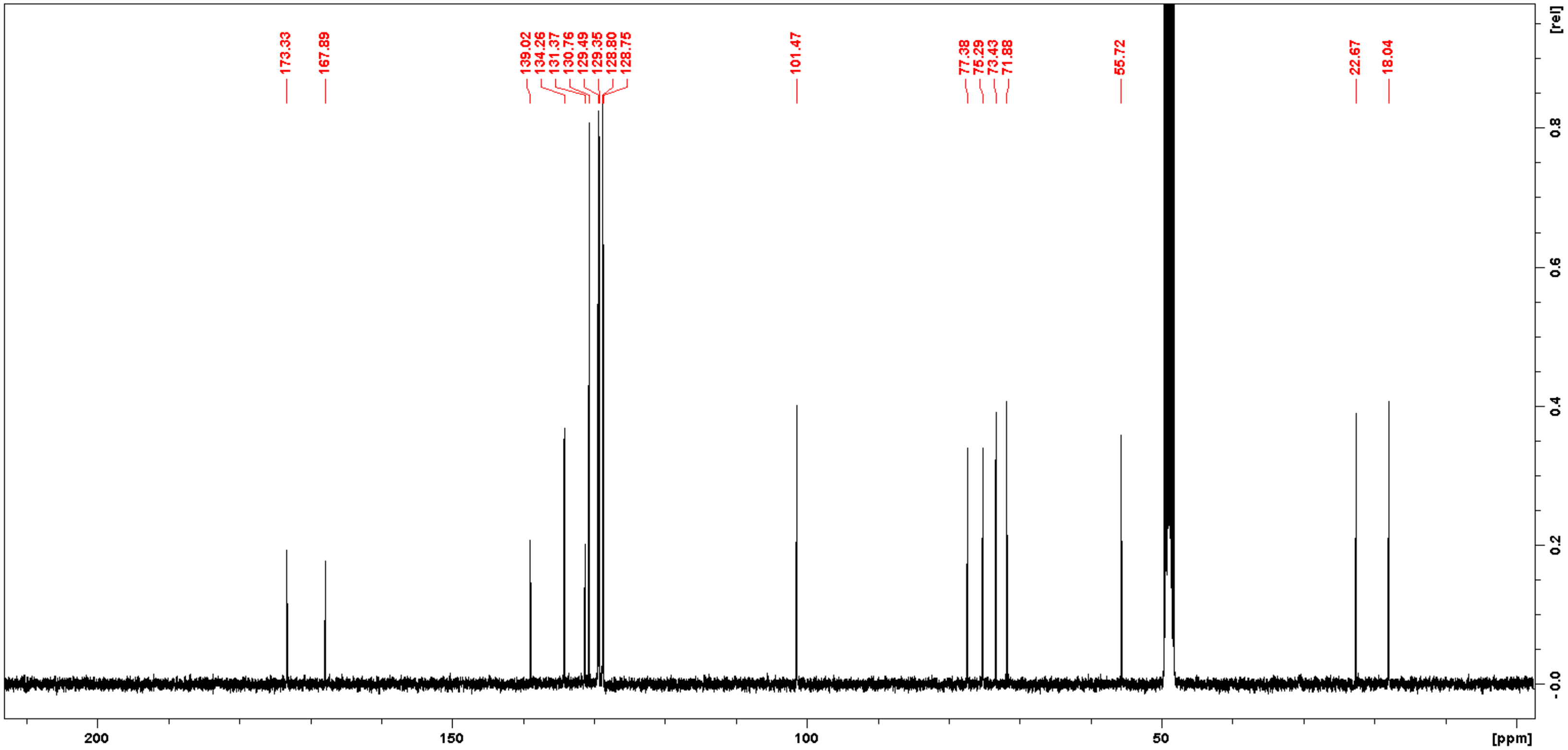

Supplementary Figure 15: 3-Benzoyl-benzyl-2-acetamido-2,6-dideoxy-β-D-glucopyranoside (6) COSY

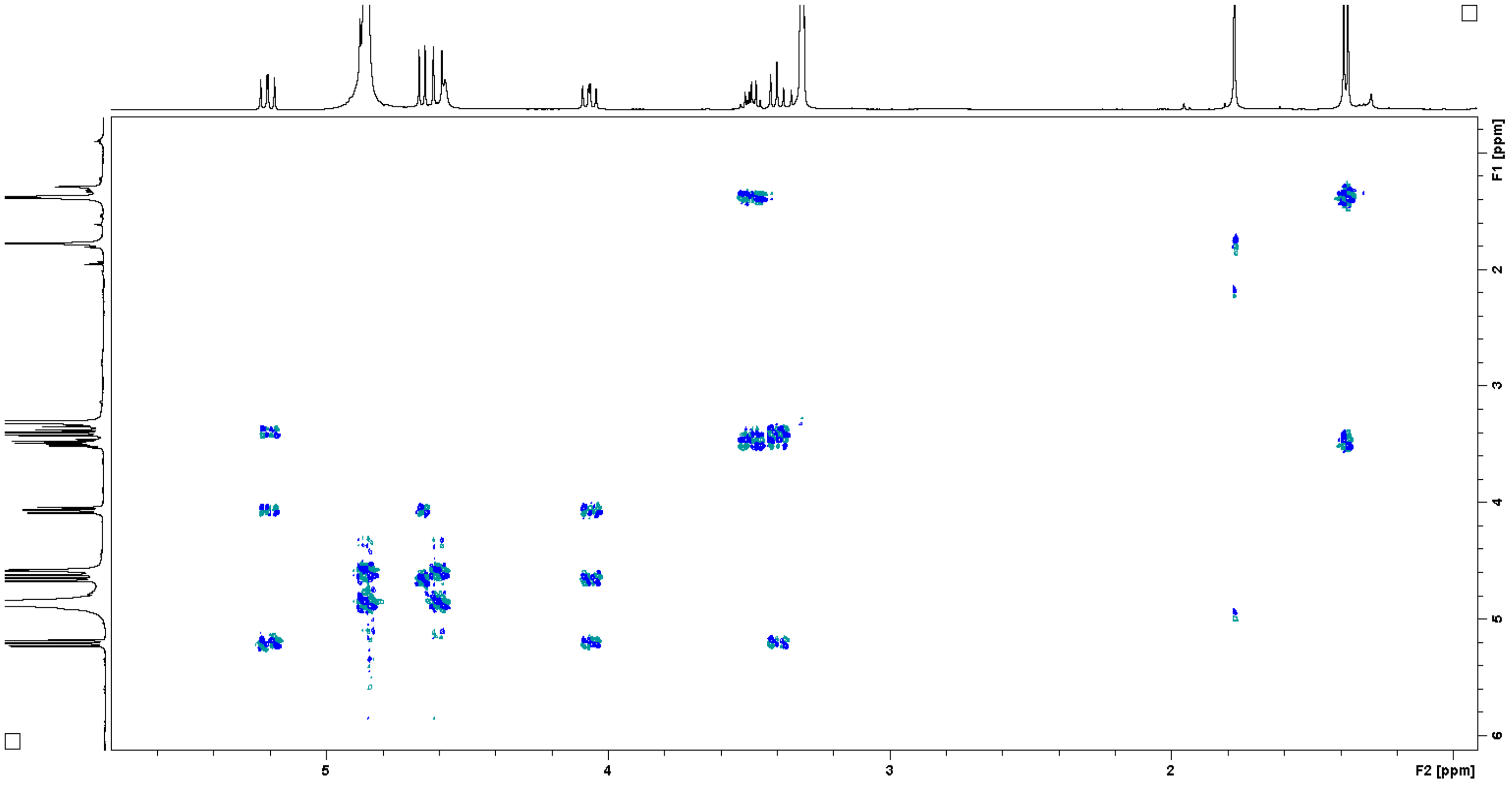

Supplementary Figure 16: 3-Benzoyl-benzyl-2-acetamido-2,6-dideoxy-β-D-glucopyranoside (6) HMBC

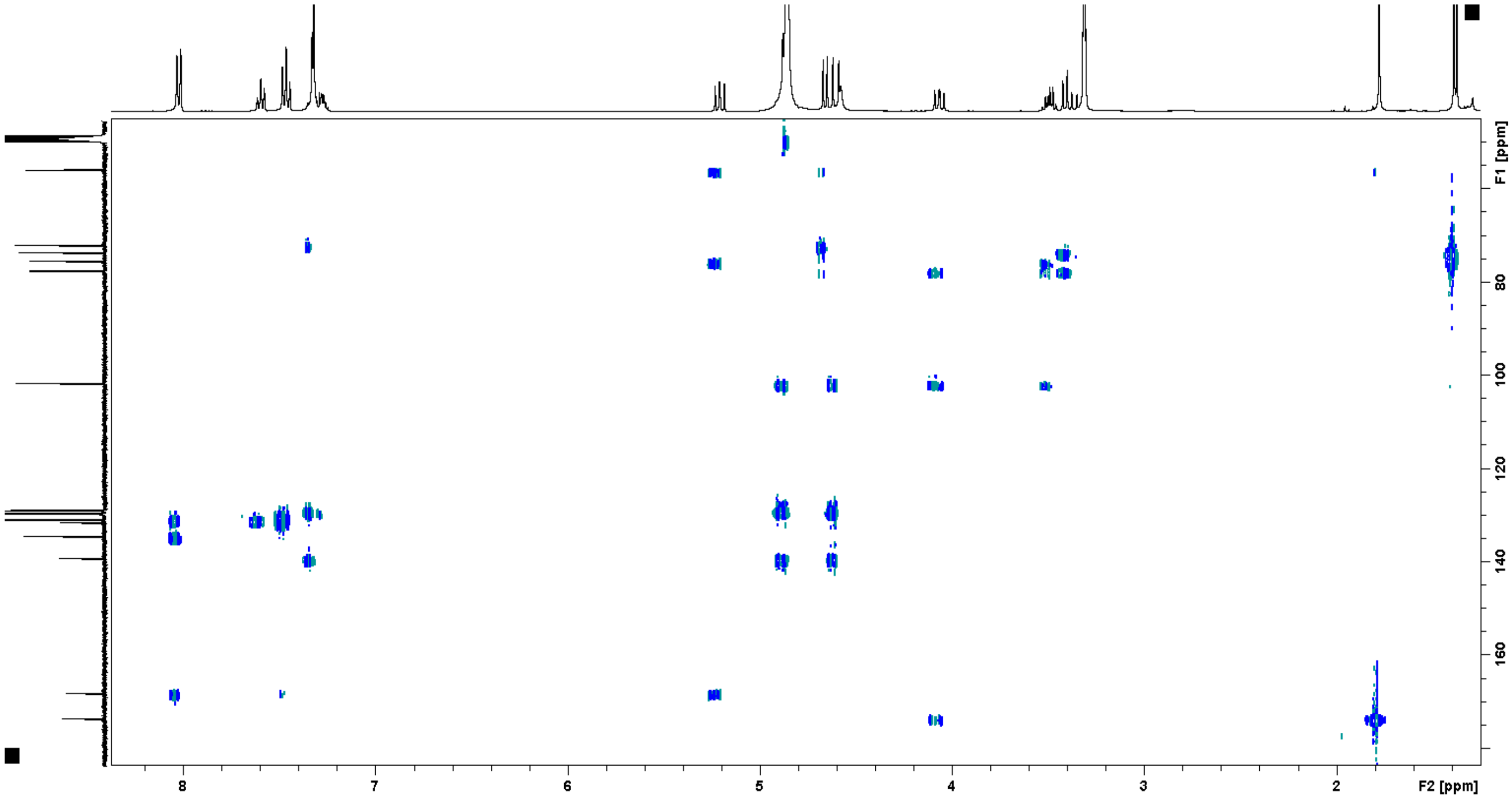

Supplementary Figure 17: benzyl 3,4,6-tri-O-acetyl-2-deoxy-2-[(2,2,2-trichloroethoxy)carbonyl]-amino-β-D-glucopyranosyl-(1$\rightarrow$ 4)-2-acetamido-3-O-benzoyl-2,6-dideoxy-β -D-glucopyranoside (8) ^{1}H NMR

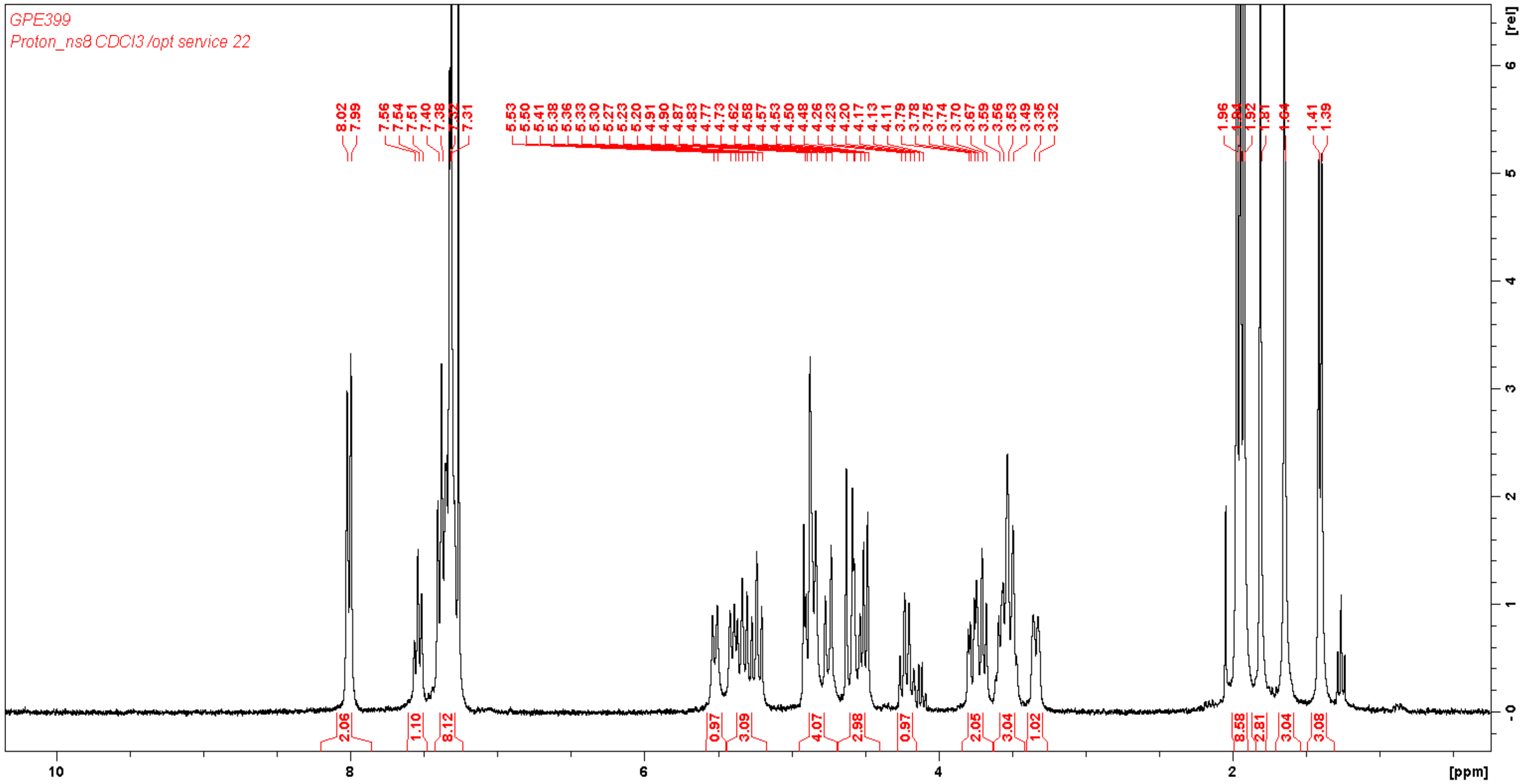

Supplementary Figure 18: benzyl 3,4,6-tri-O-acetyl-2-deoxy-2-[(2,2,2-trichloroethoxy)carbonyl]-amino-β -D-glucopyranosyl-(1$\rightarrow$ 4)-2-acetamido-3-O-benzoyl-2,6-dideoxy-β -D-glucopyranoside (8) ^{13}C NMR

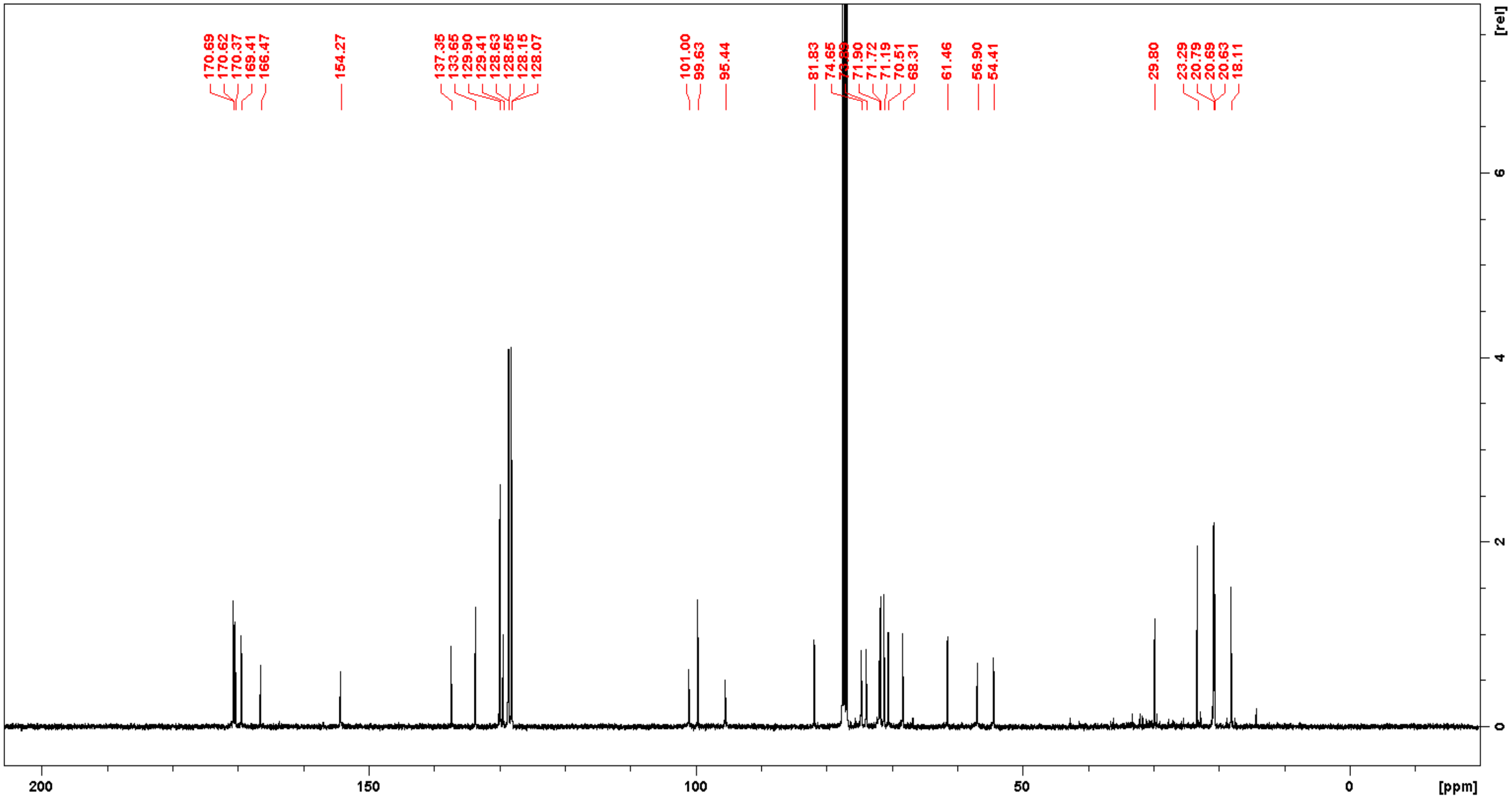

Supplementary Figure 19: benzyl 3,4,6-tri-O-acetyl-2-deoxy-2-N-acetoammido-D-glucopyranosyl-β-(1$\rightarrow$ 4)-2-acetamido-3-O-benzoyl-2,6-dideoxy β -D-glucopyranoside (9) ^{1}H NMR

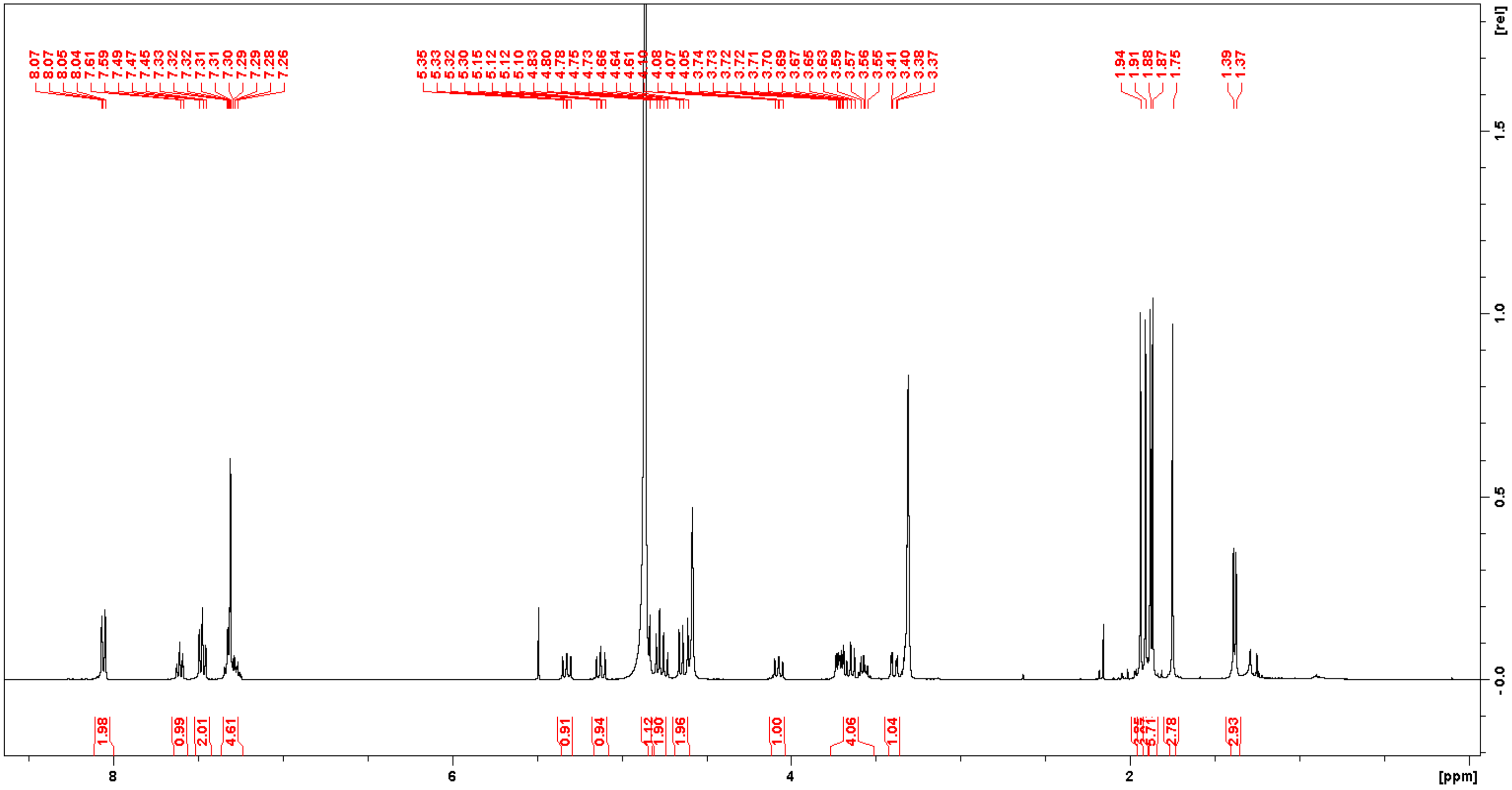

Supplementary Figure 20: benzyl 3,4,6-tri-O-acetyl-2-deoxy-2-N-acetoammido-D-glucopyranosyl-β-(1$\rightarrow$ 4)-2-acetamido-3-O-benzoyl-2,6-dideoxy-β -D-glucopyranoside (9) ^{31}C NMR

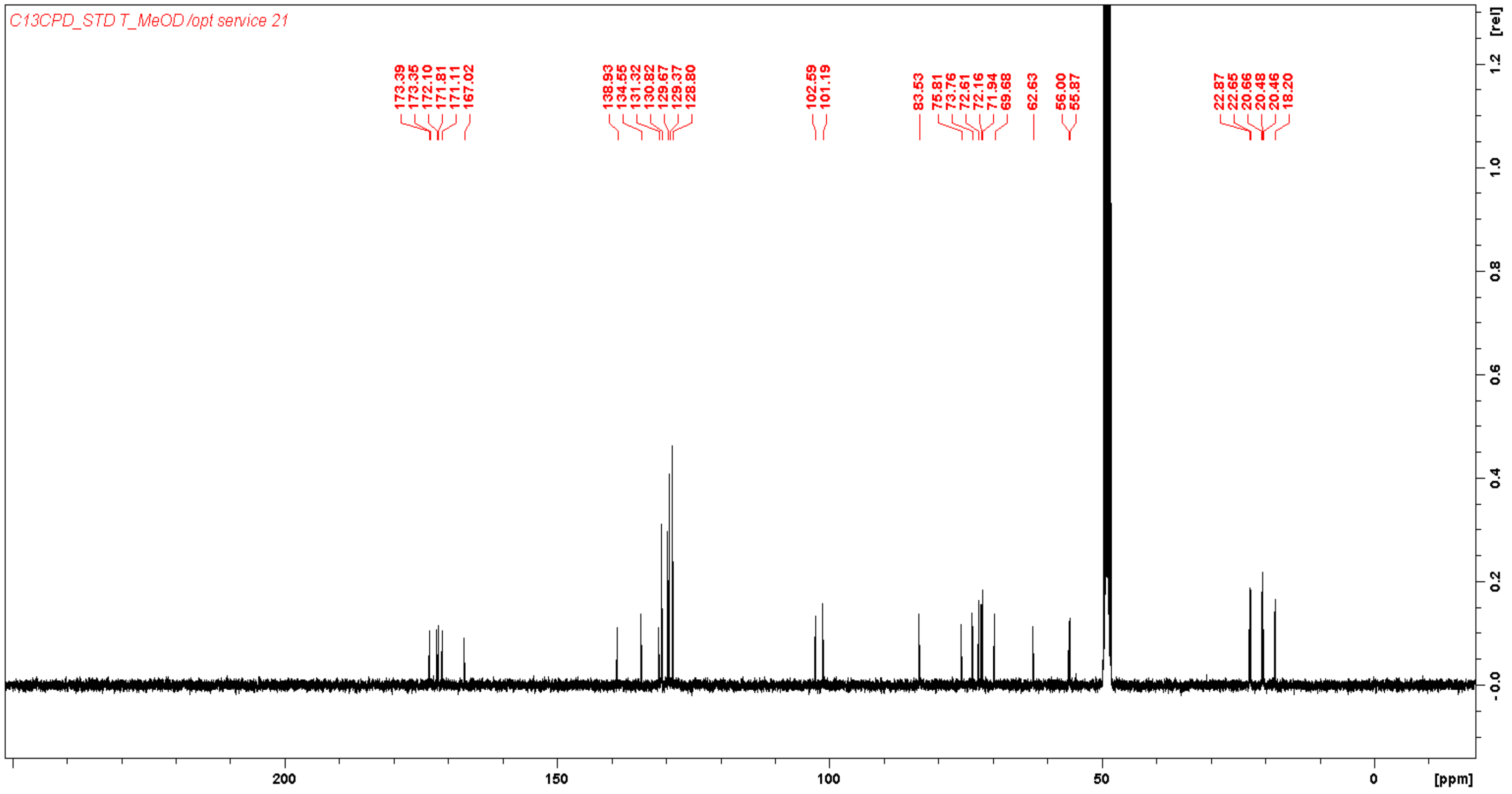

Supplementary Figure 21: benzyl 3,4,6-tri-O-acetyl-2-deoxy-2-N-acetoammido-D-glucopyranosyl-β-(1$\rightarrow$ 4)-2-acetamido-3-O-benzoyl-2,6-dideoxy-β -D-glucopyranoside (10) ^{1}H NMR

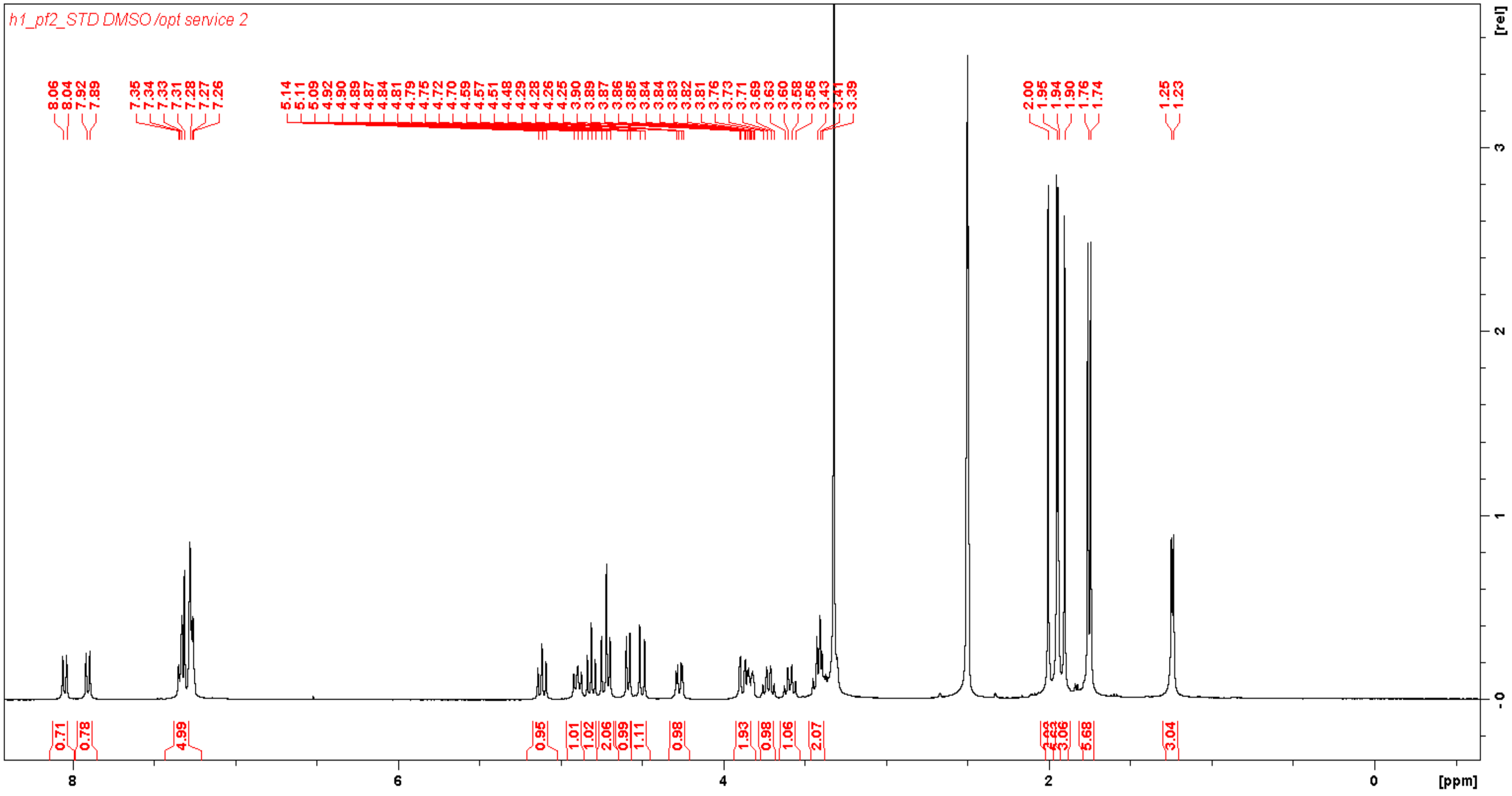

Supplementary Figure 22: benzyl 3,4,6-tri-O-acetyl-2-deoxy-2-N-acetoammido-D-glucopyranosyl-β-(1$\rightarrow$ 4)-2-acetamido-3-O-benzoyl-2,6-dideoxy-β -D-glucopyranoside (10) ^{13}C NMR

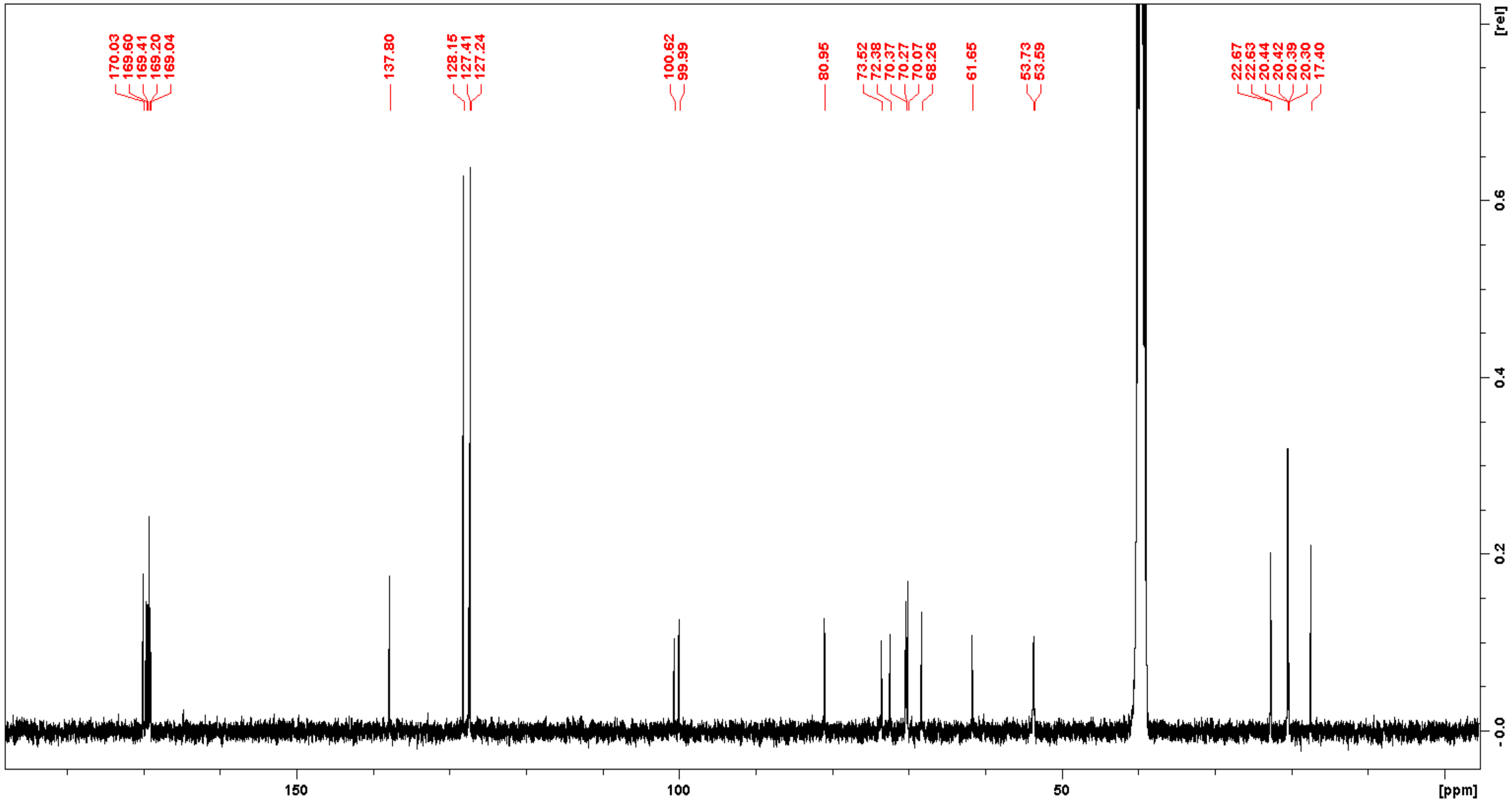

Supplementary Figure 23: 3,4,6-tri-O-acetyl-2-deoxy-2-N-acetoammido-D-glucopyranosyl-β-(1$\rightarrow$ 4)-2-acetamido-3-O-benzoyl-2,6-dideoxy-α -D-glucopyranoside-1-dibenzyl phosphate (12) ^{1}H NMR

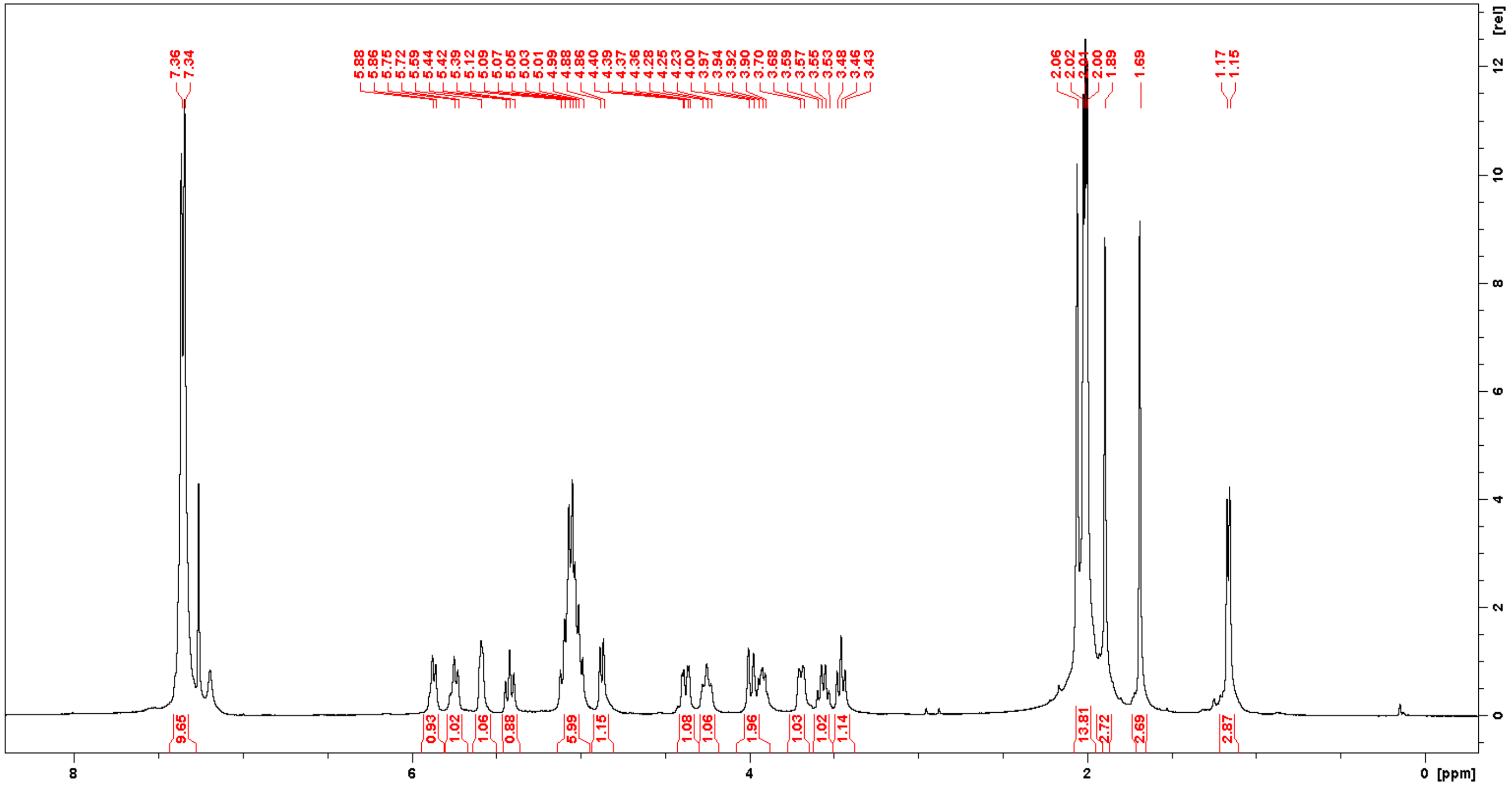

Supplementary Figure 24: 3,4,6-tri-O-acetyl-2-deoxy-2-N-acetoammido-D-glucopyranosyl-β-(1$\rightarrow$ 4)-2-acetamido-3-O-benzoyl-2,6-dideoxy-α -D-glucopyranoside-1-dibenzyl phosphate (12) ^{13}C NMR

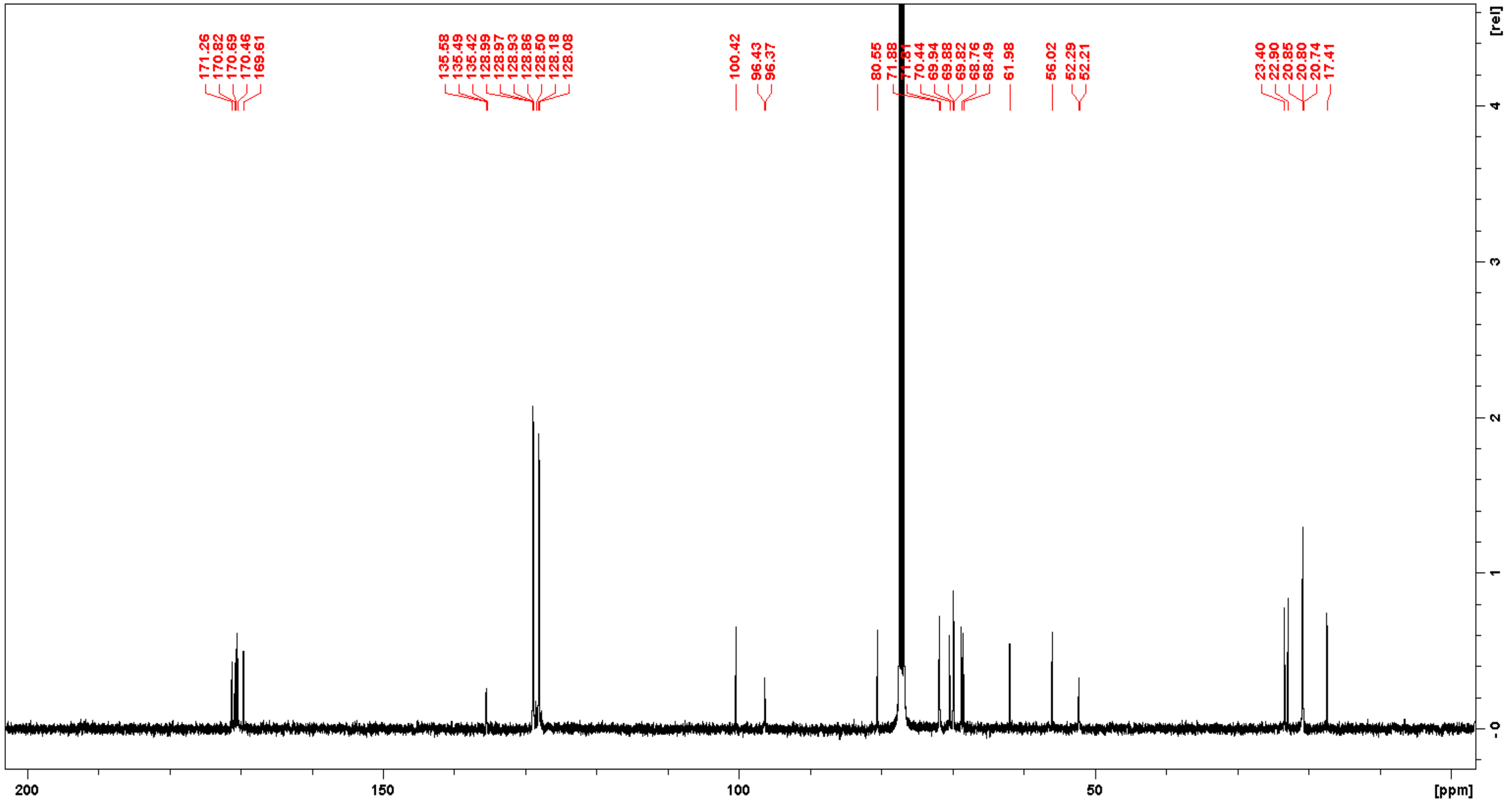

Supplementary Figure 25: 3,4,6-tri-O-acetyl-2-deoxy-2-N-acetoammido-D-glucopyranosyl-β-(1$\rightarrow$ 4)-2-acetamido-3-O-benzoyl-2,6-dideoxy-α -D-glucopyranoside-1-dibenzyl phosphate (12) ^{31}P NMR

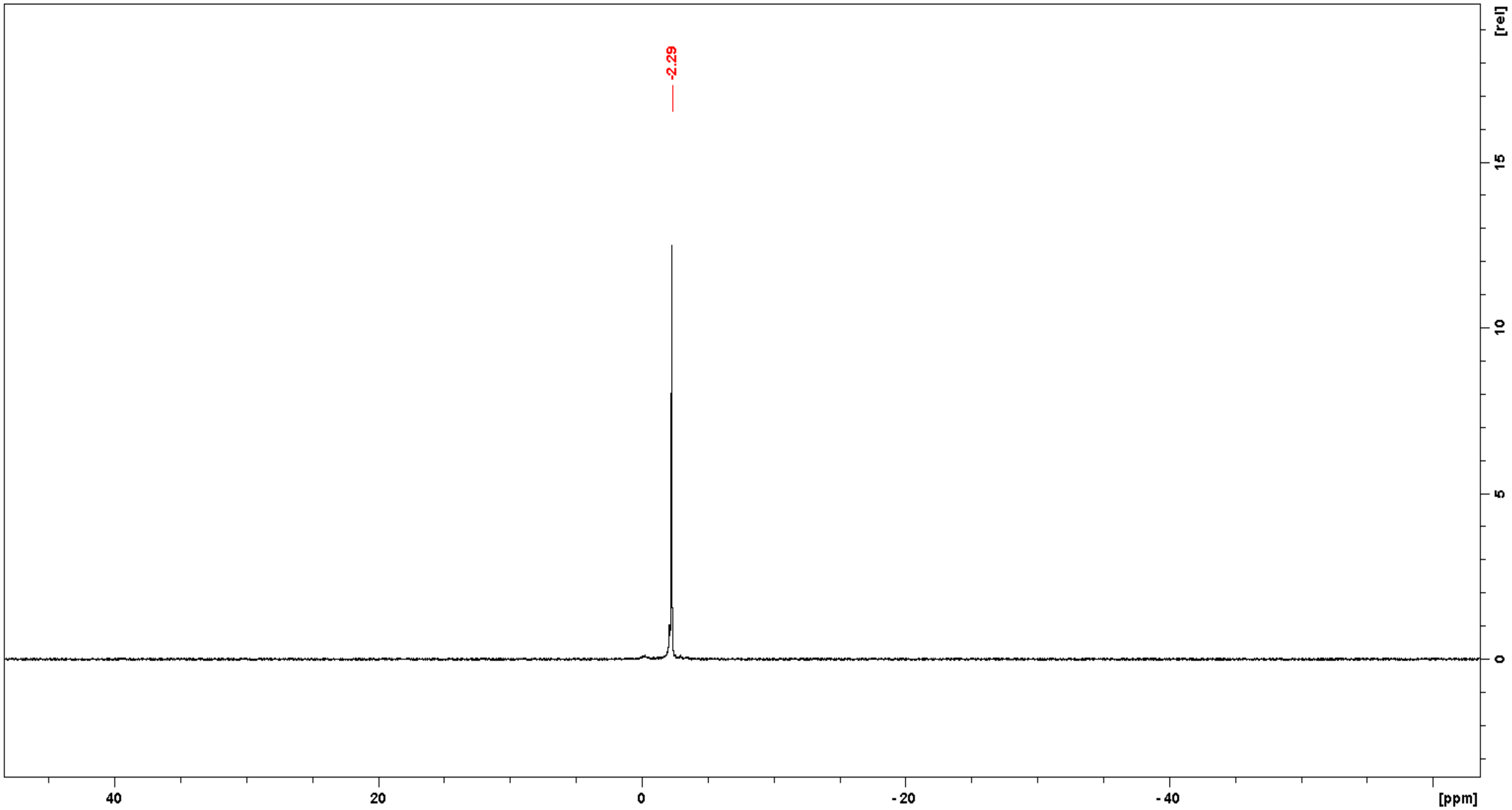

Supplementary Figure 26: 3,4,6-tri-O-acetyl-2-deoxy-2-N-acetoammido-D-glucopyranosyl-β-(1$\rightarrow$ 4)-2-acetamido-3-O-benzoyl-2,6-dideoxy-α -D-glucopyranoside-1-dibenzyl phosphate (12) COSY

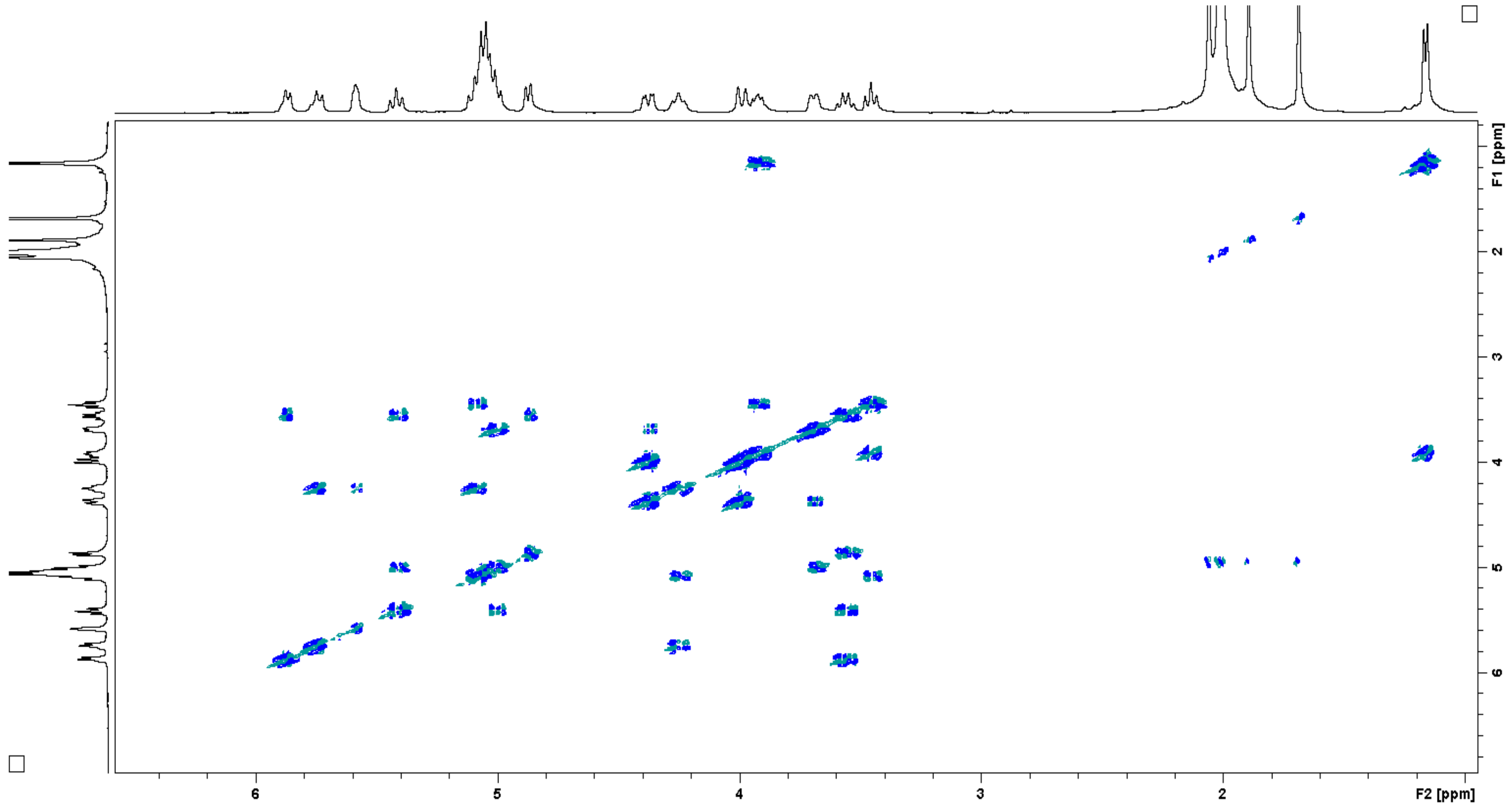

Supplementary Figure 27: Farnesylcitronellyl-pyrophosphoryl-α -3,4,6-tri-O-acetyl-2-deoxy-2-N-acetoammido-D-glucopyranosyl-β-(1$\rightarrow$ 4)-3-O-acetyl-2-acetamido-2,6-dideoxy-α -D-glucopyranoside (14) ^{1}H NMR

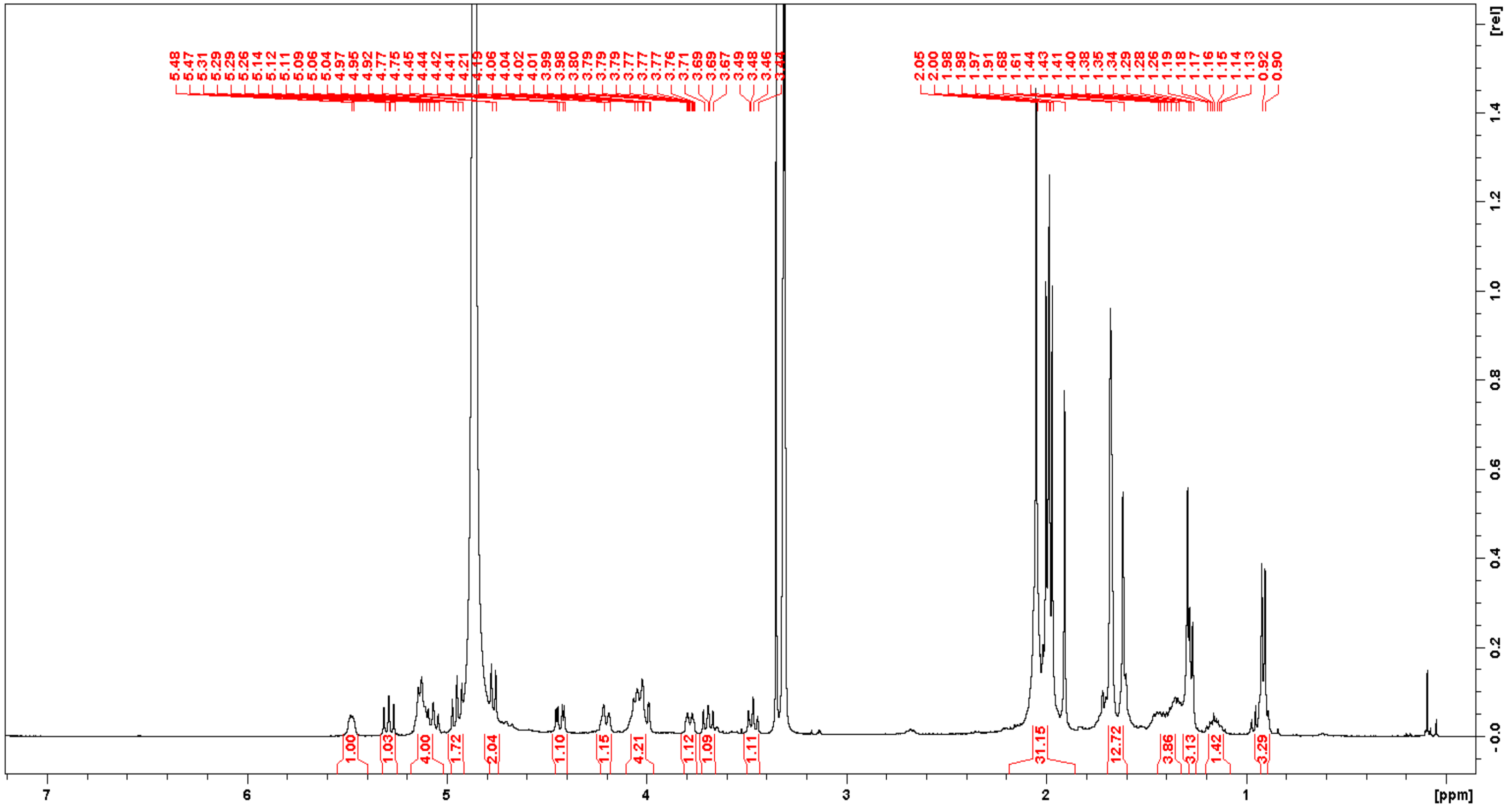

Supplementary Figure 28: Farnesylcitronellyl-pyrophosphoryl-α -3,4,6-tri-O-acetyl-2-deoxy-2-N-acetoammido-D-glucopyranosyl-β-(1$\rightarrow$ 4)-3-O-acetyl-2-acetamido-2,6-dideoxy-α -D-glucopyranoside (14) ^{13}C NMR

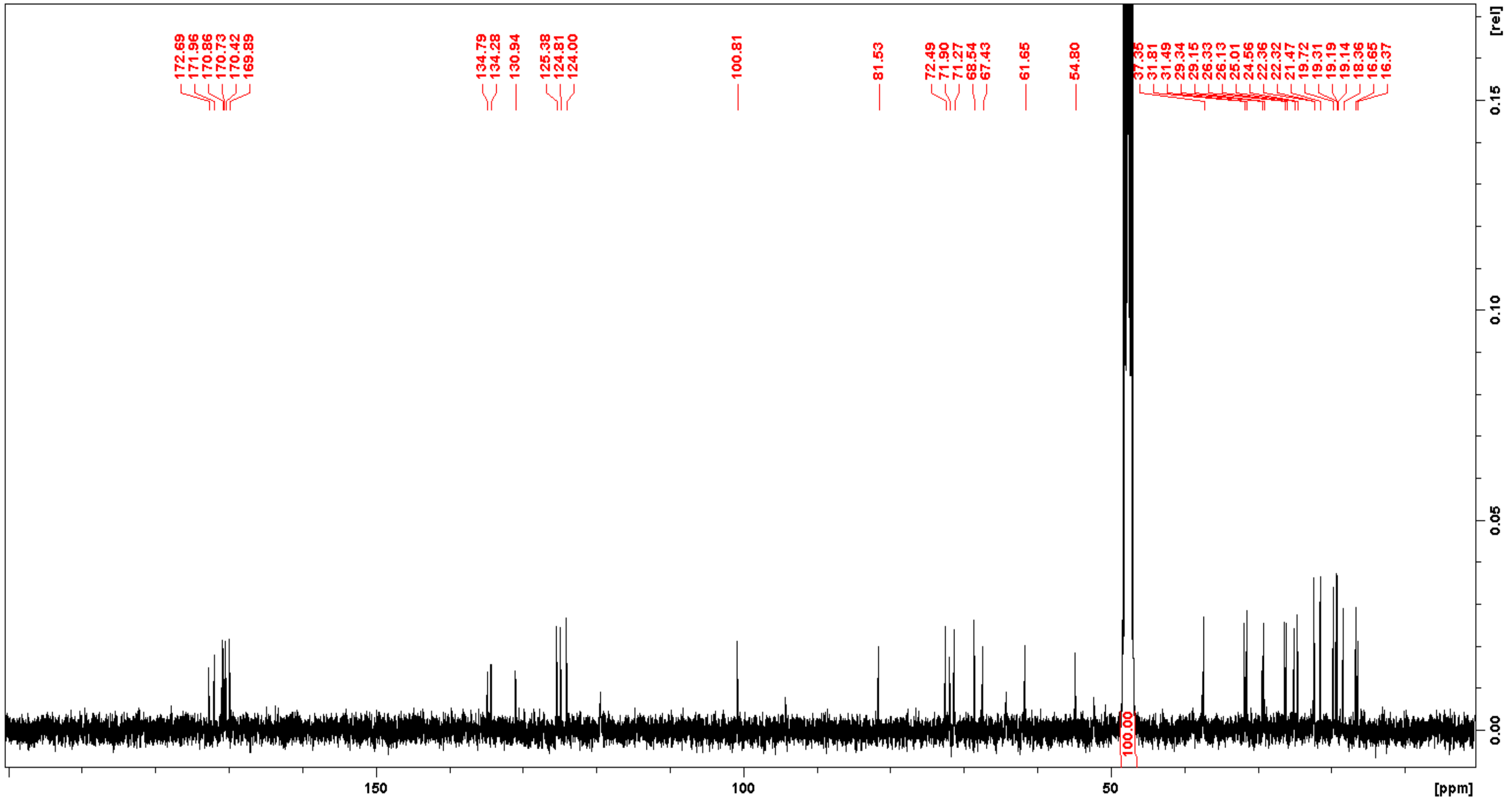

Supplementary Figure 29: Farnesylcitronellyl-pyrophosphoryl-α -3,4,6-tri-O-acetyl-2-deoxy-2-N-acetoammido-D-glucopyranosyl-β-(1$\rightarrow$ 4)-3-O-acetyl-2-acetamido-2,6-dideoxy-α -D-glucopyranoside (14) ^{31}P NMR

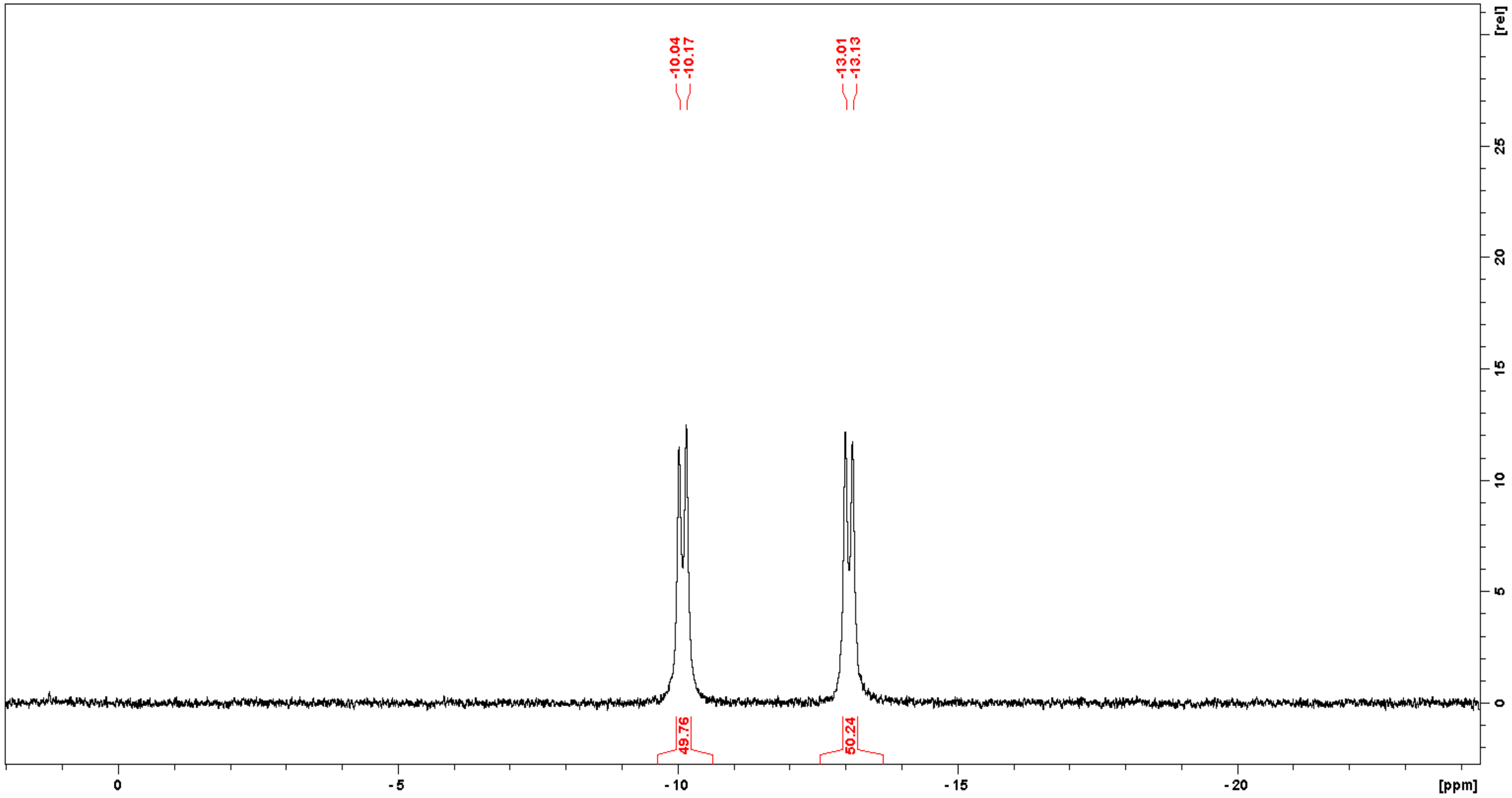

Supplementary Figure 30: Farnesylcitronellyl-pyrophosphoryl-α-2-deoxy-2-N-acetoammido-D-glucopyranosyl-β(1$\rightarrow$ 4)-2-acetamido-2,6-dideoxy-α-D-glucopyranoside (15) ^{1}H NMR

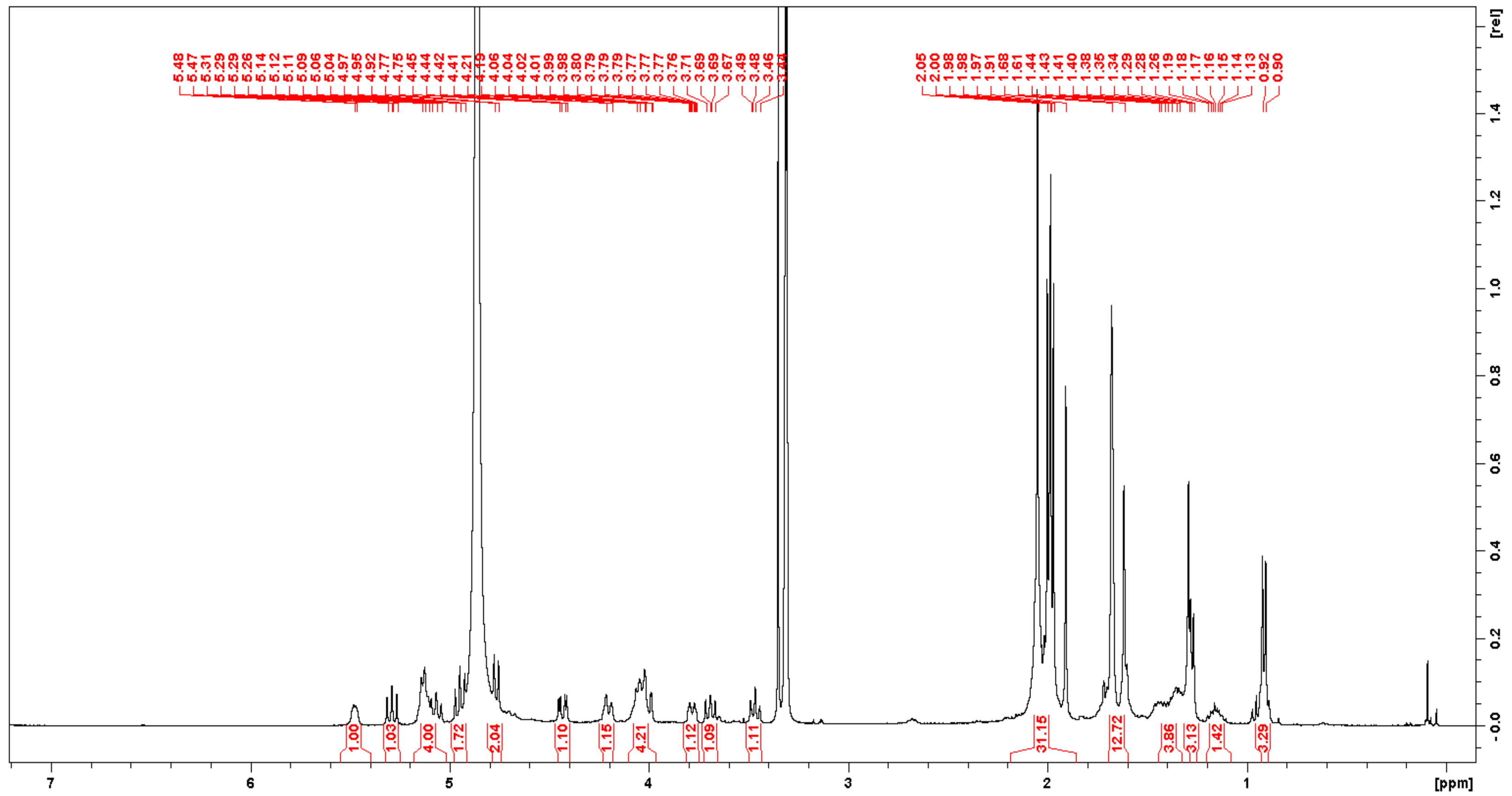

Supplementary Figure 31: Farnesylcitronellyl-pyrophosphoryl-α-2-deoxy-2-N-acetoammido-D-glucopyranosyl-β(1$\rightarrow$ 4)-2-acetamido-2,6-dideoxy-α-D-glucopyranoside (15) ^{13}C NMR

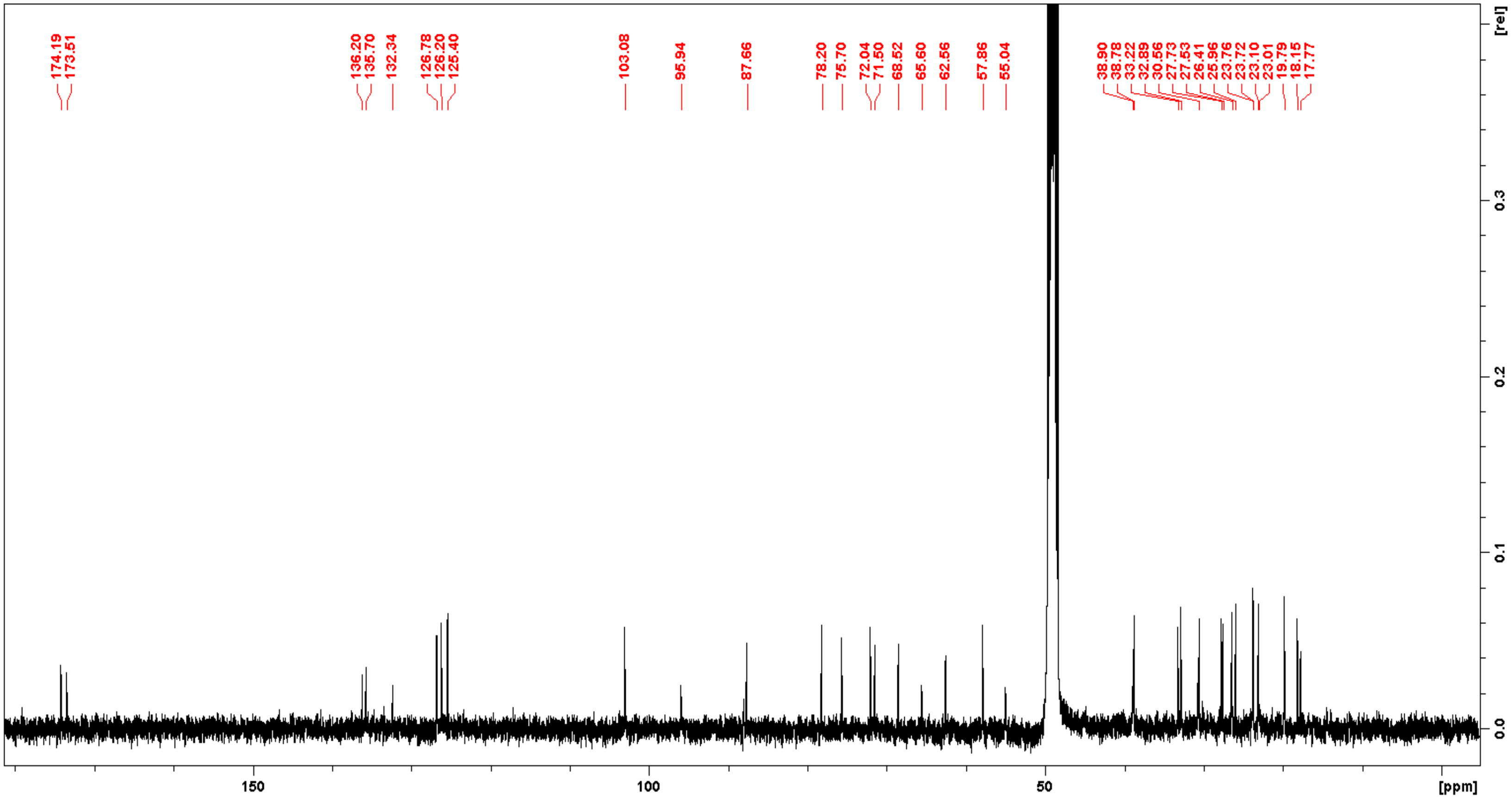

Supplementary Figure 32: Farnesylcitronellyl-pyrophosphoryl-α-2-deoxy-2-N-acetoammido-D-glucopyranosyl-β(1$\rightarrow$ 4)-2-acetamido-2,6-dideoxy-α-D-glucopyranoside (15) ^{31}P NMR

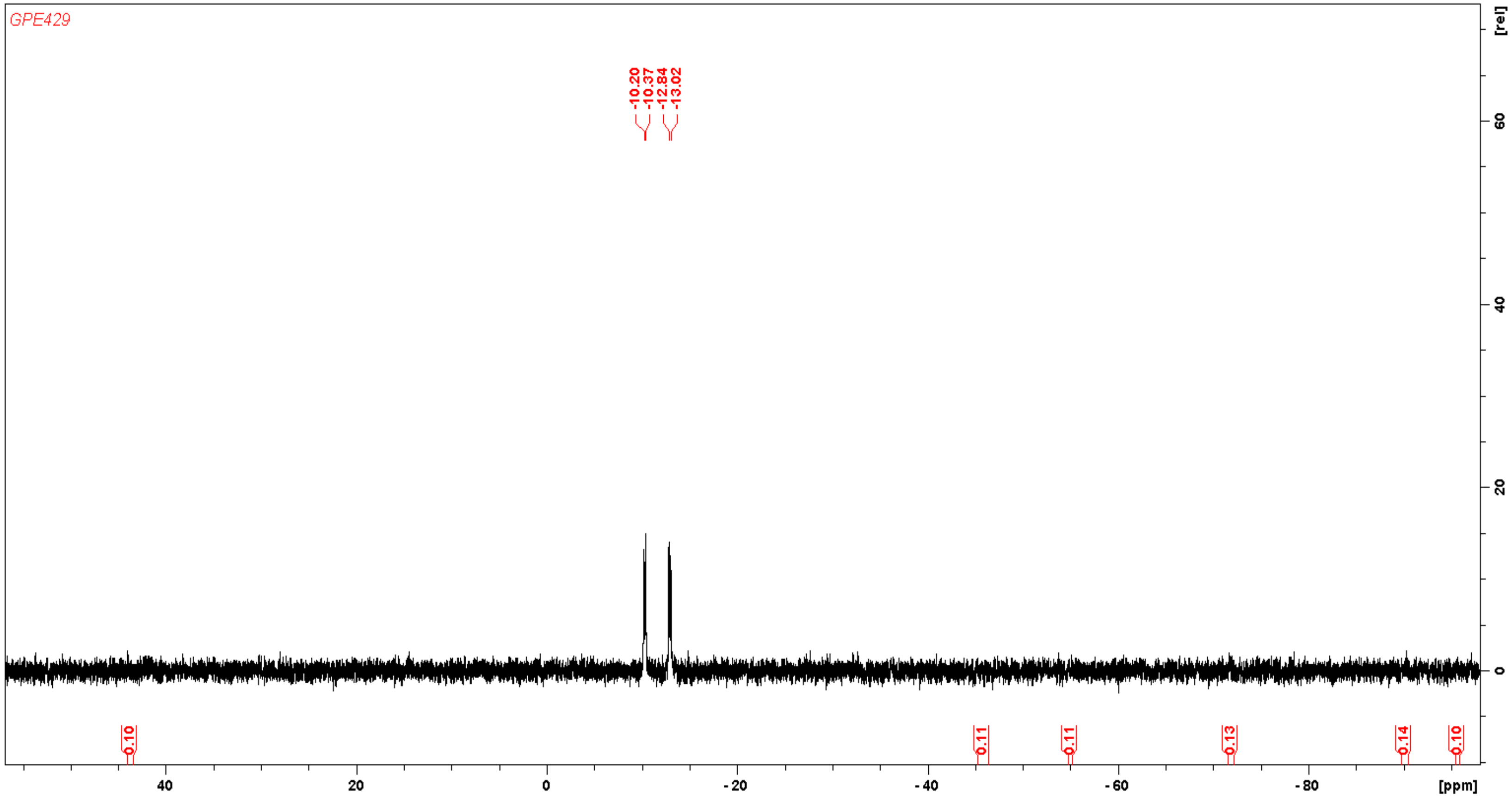

Supplementary References

(1) Lowe, D. Chemical reactions from US patents (1976-Sep2016) (2017). URL https://figshare.com/articles/Chemical_reactions_from_US_patents_1976-Sep2016_/5104873.

(2) Jin, W., Coley, C., Barzilay, R. & Jaakkola, T. Predicting organic reaction outcomes with weisfeiler-lehman network. In Guyon, I. *et al.* (eds.) *Advances in Neural Information Processing Systems 30*, 2607–2616 (Curran Associates, Inc., 2017).

(3) Schwaller, P., Gaudin, T., Lanyi, D., Bekas, C. & Laino, T. "found in translation": predicting outcomes of complex organic chemistry reactions using neural sequence-to-sequence models. *Chemical science* **9**, 6091–6098 (2018).

(4) Bradshaw, J., Kusner, M., Paige, B., Segler, M. & Hernández-Lobato, J. A generative model for electron paths. In *7th International Conference on Learning Representations, ICLR 2019* (2019).

(5) Schwaller, P. & Laino, T. Data-driven learning systems for chemical reaction prediction: An analysis of recent approaches. In *Machine Learning in Chemistry: Data-Driven Algorithms, Learning Systems, and Predictions*, 61–79 (ACS Publications, 2019).

(6) Landrum, G. *et al.* rdkit/rdkit: 2019_03_4 (q1 2019) release (2019). URL https://doi.org/10.5281/zenodo.3366468.

(7) Reaxys database. URL https://www.reaxys.com. (Accessed Oct 29, 2019).

(8) Klein, G., Kim, Y., Deng, Y., Senellart, J. & Rush, A. M. OpenNMT: Open-source toolkit for neural machine translation. In *Proc. ACL* (2017).

(9) Opennmt-py. URL https://github.com/OpenNMT/OpenNMT-py. (Accessed Oct 29, 2019).

(10) Molecular Transformer. URL `https://github.com/pschwllr/MolecularTransformer`. (Accessed Aug 29, 2019).

(11) Schwaller, P. *et al.* Molecular transformer: A model for uncertainty-calibrated chemical reaction prediction. *ACS central science* **5**, 1572–1583 (2019).

(12) Behera, A., Rai, D. & Kulkarni, S. S. Total syntheses of conjugation-ready trisaccharide repeating units of pseudomonas aeruginosa o11 and staphylococcus aureus type 5 capsular polysaccharide for vaccine development. *Journal of the American Chemical Society* **142**, 456–467 (2019).

(13) Schultz, V. L. *et al.* Chemoenzymatic synthesis of 4-fluoro-n-acetylhexosamine uridine diphosphate donors: Chain terminators in glycosaminoglycan synthesis. *The Journal of organic chemistry* **82**, 2243–2248 (2017).

(14) Dullenkopf, W., Castro-Palomino, J. C., Manzoni, L. & Schmidt, R. R. N-trichloroethoxycarbonyl-glucosamine derivatives as glycosyl donors. *Carbohydrate research* **296**, 135–147 (1996).

(15) Ramírez, A. S. *et al.* Characterization of the single-subunit oligosaccharyltransferase stt3a from trypanosoma brucei using synthetic peptides and lipid-linked oligosaccharide analogs. *Glycobiology* **27**, 525–535 (2017).
